# Chemical Fermentation PoreCreation on Multilevel Bio-Carbon Structure with In Situ Ni–Fe Alloy Loading for Superior Oxygen Evolution Reaction Electrocatalysis

**DOI:** 10.1007/s40820-025-01777-2

**Published:** 2025-05-21

**Authors:** Qiaoling Kang, Mengfei Su, Yana Luo, Ting Wang, Feng Gao, Qingyi Lu

**Affiliations:** 1 State Key Laboratory of Coordination Chemistry, Collaborative Innovation Center of Advanced Microstructures, School of Chemistry and Chemical Engineering, Coordination Chemistry Institute, Nanjing University, Nanjing, 210023 People’s Republic of China; 2 Department of Materials Science and Engineering, Jiangsu Key Laboratory of Artificial Functional Materials, Collaborative Innovation Center of Advanced Microstructures, College of Engineering and Applied Sciences, Nanjing University, Nanjing, 210093 People’s Republic of China; 3 College of Materials and Chemistry, China Jiliang University, Hangzhou, 310018 People’s Republic of China

**Keywords:** Ni–Fe alloys, Multilevel porous network, Chemical fermentation pore creation, Ultra-low overpotential, Oxygen evolution reaction electrocatalysts

## Abstract

In the quest for high-efficiency and cost-effective catalysts for the oxygen evolution reaction (OER), a novel biomass-driven strategy is developed to fabricate a unique one-dimensional rod-arrays@two-dimensional interlaced-sheets (C_1D@2D_) network. A groundbreaking chemical fermentation (CF) pore-generation mechanism, proposed for the first time for creating nanopores within carbon structures, is based on the optimal balance between gasification and solidification. This mechanism not only results in a distinctive C_1D@2D_ multilevel network with nanoscale, intersecting and freely flowing channels but also introduces a novel concept for in situ, extensive and hierarchical pore formation. The unique architecture, combined with the homogeneous dispersion of Ni–Fe nanoparticles, facilitates easy electrolyte penetration and provides abundant active sites for the anchoring and dispersion of reactive molecules or ions. Consequently, the Ni–Fe@C_1D@2D_ porous network demonstrates an exceptional OER electrocatalytic performance, achieving a record-low overpotential of 165 mV at 10 mA cm^−2^ and maintaining long-term stability for over 90 h. Theoretical calculations reveal that the porous structure markedly strengthens the interaction between alloy nanoparticles and the carbon matrix, thereby significantly boosting their electrocatalytic activity and stability. These findings unequivocally validate the CF pore-generation mechanism as a powerful and innovative strategy for designing highly efficient functional nanostructures.

**Supplementary Information:**

The online version contains supplementary material available at 10.1007/s40820-025-01777-2.

## Highlights

- A groundbreaking chemical fermentation pore-generation mechanism is developed for the first time for creating nanopores within carbon structures to form multilevel porous network based on the optimal balance between gasification and solidification.
- The Ni–Fe@C_1D@2D_ porous network demonstrates an exceptional oxygen evolution reaction electrocatalytic performance, achieving an ultralow overpotential of 165 mV at 10 mA cm^−2^ on a non-supported inert electrode and maintaining long-term stability for over 90 h.

**Supplementary Information:**

The online version contains supplementary material available at 10.1007/s40820-025-01777-2.

## Graphical abstract

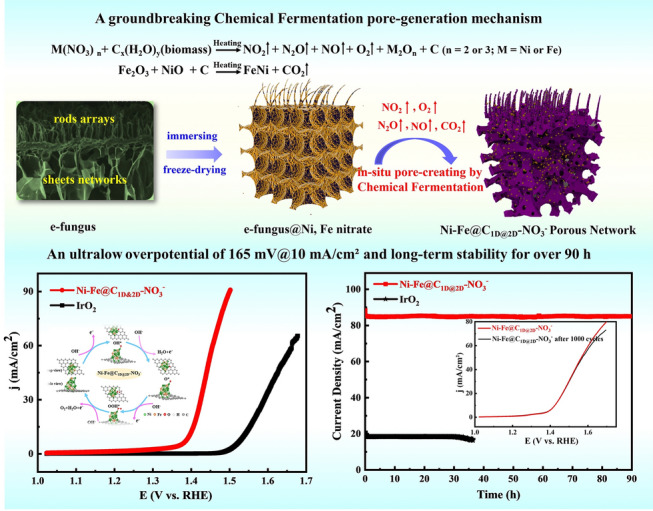

## Introduction

Environmental pollution and climate issues stemming from oil consumption, along with energy crisis, have stimulated intensive researches on the renewable energy technologies [1–3]. Hydrogen energy, as a clean, efficient and reproducible energy, has become an attractive measure for solving the energy supply security and the greenhouse gas reduction [4–6]. Water electrolysis, a promising hydrogen-producing technology on large scale, involves hydrogen evolution reaction (HER) and oxygen evolution reaction (OER), in which the latter is the kinetically rate-limiting step owing to its four-electron-transfer process [7, 8]. Thus, exploring highly efficient OER electrocatalysts then becomes a significant demand [9–11].

Overpotential (η) is one of the most critical parameters to evaluate electrocatalysts’ OER activity, and a lot of efforts have been made to synthesize electrocatalysts with low overpotential. Loading active substance on conductive substrate is considered to be beneficial to reduce the overpotential due to high catalyst loading and low electric conductivity. For examples, overpotentials of 154 mV at 10 mA cm^−2^ have been achieved by Ni foam electrodes with loading of amorphous (Ni, Fe)OOH or FeP/Ni_2_P [12, 13]. However, loading the active materials onto the foam substrate is usually difficult or fussy, not universal for most of the electrocatalysts, which limits its application scope. More non-supported catalysts have attracted lots of efforts [14–16]. However, the decrease of overpotential is quite difficult and the reported overpotential is usually higher than 200 mV at 10 mA cm^−2^. Very recently, Qin’s group reported a novel fabrication of mesoporous single crystal with lots of active edges around the mesopores by Fe modification and achieved an ultralow η of 185 mV at 10 mA cm^−2^ [17]. No electrocatalysts on non-supported inert electrode with overpotential less than 180 mV at 10 mA cm^−2^ have been reported. Constructing special structures to further decrease overpotential is still a research focus, but proved to be a big challenge so far.

Recently, bimetallic alloy catalysts, such as NiFe, NiCo and FeCo, exhibit superior activity in comparison to their individual constituents, probably because the co-existence of two different metals can adjust the electronic structure each other forming synergistic effect to promote catalytic reactions [18–20]. Among the miscellaneous bimetallic alloys, Ni–Fe alloy reveals great application potentials because nickel and iron are both environmentally friendly and earth abundant and have changes in valence state, which is especially significant for OER [21]. Hybridizing the catalysts with carbon materials has also been employed to enhance the conductivity of bimetallic alloy catalysts [22]. The used carbon materials including graphene, carbon nanotube and carbon nanofiber are beneficial to OER [23, 24], but with high costs and/or complex preparation processes. Moreover, such carbon structures are not helpful for the permeation of the electrolyte and delay the further reaction. So far, the lowest reported overpotential with such bimetallic alloy@carbon catalysts is about 226 mV at 10 mA cm^−2^ [25].

To achieve high catalytic efficiency, an ideal catalyst structure might possess enough open, interconnected and hierarchical pores to accommodate products, avoid electrode clogging and facilitate electrolyte permeation. Interlaced three-dimensional (3D) structure with high conductivity and numerous homodisperse active sites also helps to increase catalytic activity. Biomasses always possess well-defined morphologies, wonderful micro-/nanostructures and unique naturally occurring pores and are ideal precursors to synthesize nanocarbon materials with exquisite complex architectures [26, 27]. Natural edible fungus (e-fungus) has a special 3D ordered mesoporous nanostructure including neat rod-arrays on the top and interlaced-sheets network at the bottom, leading to high water absorption, good water retention and uniform water dispersion, which makes it very suitable for high-efficient catalyst. Herein, we propose a biomass-driven strategy to construct a special 3D multilevel carbon network (carbon (1D) rod-arrays@(2D) interlaced-sheets, C_1D@2D_) with open, interconnected and hierarchical porous structure and monodispersed Ni–Fe alloy nanoparticles loading (Ni–Fe@C_1D@2D_ porous network). The multilevel crisscrossed carbon network, inherited from e-fungus, enhances the catalyst’s electric conductivity. Its space geometrical structure, better maintained through freeze-drying than conventional oven-drying, greatly increases the catalyst’s accommodation space. Moreover, a brand-new chemical fermentation (CF) pore-creating mechanism is proposed for the first time for the creation of a huge number of nanopores, leading to a special multilevel carbon network full of nanosized crisscrossing channels. This distinct porous structure benefits the electrolyte permeation to accelerate the mass transfer rate of electrode reactions. The simultaneous loading of monodispersed Ni–Fe alloy nanoparticles offers more active sites for active molecules or ions to anchor and disperse, further speeding up the catalytic reaction. As a result, Ni–Fe@C_1D@2D_ porous network exhibits an unprecedented high electrocatalytic activity for OER with the lowest overpotential on non-supported inert electrode (165 mV at 10 mA cm^−2^ vs RHE). Its high catalytic activity, together with a long-term stability of more than 90 h, far surpasses the performances of current OER electrocatalysts reported to date. Theoretical calculations indicate that the porous structure of the carbon would significantly enhance the interaction between Ni-Fe alloy nanoparticles and carbon matrix to boost their electrocatalytic activity and stability. This work not only brings up a simple and universal biomass-promoted technique for high-activity catalysis, but also proposes a brand-new CF pore-creating concept as an effective structure-promoting strategy for high-performance functional nanostructures design.

## Experimental Section

### Materials

Raw agaric was purchased from Yonghui Supermarket, Nanjing. Nickel nitrate (Ni(NO_3_)_2_·6H_2_O), ferric nitrate (Fe(NO_3_)_2_·9H_2_O), nickel acetate (Ni(OOCCH_3_)_2_·4H_2_O), ferric acetate (Fe(OOCCH_3_)_2_), nickel chloride(NiCl_2_·6H_2_O), ferric chloride (FeCl_3_·6H_2_O) and high-purity nitrogen were purchased from Sinopharm Chemical Reagent Co. Ltd, and Nanjing Shangyuan Industrial Gas Plant, respectively. Acetic acid (CH_3_COOH), hydrochloric acid (HCl) or nitric acid (HNO_3_) aqueous solution was purchased from XILONG Scientific Co. Ltd. All the reagents were used as received.

### Synthesis of Ni–Fe@C_1D&2D_-NO_3_^−^ Porous Networks

Typically, e-fungus was rinsed several times with deionized water, absolute ethanol and 0.5 M H_2_SO_4_ and dried at 60 °C in oven. Then, 1 g of e-fungus was immersed in 200 mL mixture of 0.075 M Ni(NO_3_)_2_·6H_2_O and 0.025 M Fe(NO_3_)_2_·9H_2_O aqueous solution with Ni/Fe molar ratio of (1:0, 6:1, 3:1, 1:3, 1:6 and 0:1) under vigorous magnetic stirring for 24 h at room temperature. The as-obtained e-fungus with absorbed aqueous solution was frozen in liquid nitrogen (-196 °C) and freeze-dried in a bulk tray dryer at a sublimation temperature of − 82 °C and a pressure of 0.04 mbar to evaporate water. Finally, the dried e-fungus/nitrate hybrid was pyrolyzed under N_2_ flowing at 700 °C for 3 h to generate black Ni-Fe@C_1D&2D_-NO_3_^−^. For comparisons, through the similar procedure, Ni–Fe@C_1D&2D_-CH_3_COO^−^ and Ni–Fe@C_1D&2D_-Cl^−^ were prepared by substituting acetates or chlorides aqueous solutions for the nitrate solution, and C_1D&2D_-H_2_O, C_1D&2D_-CH_3_COO^−^, C_1D&2D_-Cl^−^ and C_1D&2D_-NO_3_^−^ were prepared by immersing e-fungus in distilled water, 0.5 M CH_3_COOH, 0.5 M HCl or 0.5 M HNO_3_ aqueous solution.

### Characterizations

X-ray diffraction (XRD) patterns of the products were collected on a Bruker D8 ADVANCE diffractometer with Cu K_α_ radiation (*λ*= 1.5418 Ǻ). The structures and morphologies of the samples were observed by scanning electron microscopy (SEM, Hitachi S-4800) and transmission electron microscope (TEM, JEM 2800). An energy-dispersive X-ray spectroscope (EDS) attached to the SEM was used to analyze the composition of the samples. In situ Raman spectra were collected with a Horiba LabRAM Aramis spectrometer using a laser with a wavelength of 532 nm as the excitation source with homemade reactor. X-ray photoelectron spectroscopy (XPS) analyses were conducted on a PHI-5000 Versa Probe X-ray photoelectron spectrometer using an Al Kα X-ray source. N_2_ sorption analyses were conducted using an ASAP 2020 accelerated surface area and a porosimetry instrument (Micromeritics, Norcross, GA, USA), equipped with an automated surface area, at 77 K using Barrett–Emmett–Teller (BET) calculations for the surface area and Barrett–Joyner–Hanlenda for pore size distribution. Contact angles (CAs) were measured with different liquids on a video optical contact angle system (OCA 20, Data Physics, Germany) at room temperature. All the CAs were determined by averaging values measured at 6 different points on each sample surface. Fourier transform infrared (FT-IR) spectra were measured on a Bruker TENSOR 27 IR spectrometer. Ion chromatography (ICS-5000) was used to test the NO_3_^−^. Thermal analysis: TGA-MS was carried out on a STA8000-Spectrum 3-Clarus690 SQ8C instruments. Ten milligrams of powder was loaded for each experiment to measure mass loss with increasing temperature. Helium gas was used as the carrier gas for all experiments. The temperature was ramped from room temperature to 800 °C at a 10 °C min^−1^ scan rate. The composition of exhaust during heating was analyzed by a STA8000-Spectrum 3-Clarus690 SQ8C mass spectrometer.

### Electrocatalytic OER Measurements

The electrochemical measurements were performed in a conventional three-electrode cell using a CHI760D (Shanghai Chenhua, China) electrochemical workstation at room temperature with 3.0-mm-diameter catalyst-coated glassy carbon as the working electrode, a graphite rod as the counter electrode and a Hg/HgO electrode as the reference electrode, respectively. The electrolyte was purged with N_2_ for 1 h to remove the dissolved gases completely before the electrochemical measurements. Note that the current density was normalized to the geometrical area and all potentials measured were calibrated to the reversible hydrogen electrode (RHE) according to Eq. (1):

1 $$E_{{{\text{vs}}\,{\text{RHE}}}} = E_{{{\text{vs}}\,{\text{Hg}}/{\text{HgO}}}} + 0.098 + 0.059\,{\text{pH}}$$

The catalyst ink was prepared by blending 5 mg of each catalyst with 40 μL of Nafion solution (4 wt%) and 960 μL of water/isopropanol solution (3:1) via sonication. An amount of 10 μL of the dispersion was transferred onto the glassy carbon electrode with a catalyst loading of about 0.71 mg cm^−2^. Then, the as-prepared catalyst electrode was dried at room temperature. Using this electrode as the working electrode, electrochemical OER measurements were conducted in 1 M KOH solution. Polarization curves were obtained at a scan rate of 5 mV s^−1^. Accelerated degradation measurement was conducted for 1000 cyclic voltammetry (CV) cycles in 1 M KOH solution at a scan rate of 50 mV s^−1^. Electrochemical impedance spectroscopy (EIS) was performed with frequency range of 0.01–100 kHz at a bias potential of 1.5 V (vs. RHE) by the Zahner IM6EX workstation. The ohmic potential drop (i-R) losses that arise from the solution resistance were all corrected through the EIS technique. The potentiostat has a frequency range of 10 kHz-100 MHz in a 10.0 mV AC voltage. In all measurements, the reference electrode was calibrated with respect to reversible hydrogen electrode (RHE). All polarization data were without iR-corrected. RRDE voltammetry experiments were performed using a CHI760D (Shanghai Chenhua, China), a speed control unit (Princeton Applied Research Model 636 Electrode Rotator with the rotating speed of the RRDE held at 1600 r.p.m.) and a PINE RRDE with a glassy carbon (GC) disk and Pt ring. The three-electrode cell consisted of a Hg/HgO electrode as the reference electrode, a graphite rod as the counter electrode and a GC electrode (5.61 mm in diameter for the RRDE test) modified by catalysts as the working electrode. For determination of the OER reaction pathway by detecting the HO_2_^−^ formation, the ring potential was held at 1.42 V vs. RHE with a rotation rate of 1600 r.p.m. in a O_2_-saturated 1 M KOH solution. To calculate the Faradaic efficiency (FE) of the system, the ring potential was held at 0.4 V vs. RHE in a N_2_-saturated 1 M KOH solution with a rotation rate of 1600 r.p.m. The FE was calculated by Eq. (2):

2 $${\text{FE}} = \frac{{I_{{{\text{ring}}}} }}{{I_{{{\text{disk}}}} \times C_{e} }} \times 100\%$$

where *I*_ring_ is the collection current on the Pt ring electrode, *I*_disk_ is the given current on the disk electrode and *C*_*e*_ is the oxygen collection coefficient (25.6%) for this type of electrode configuration.

### Theory Calculations

Our calculations were performed based on density functional theory (DFT) implemented in the QUANTUM-ESPRESSO package. Structural relaxation was performed by using spin-polarized scalar relativistic ultrasoft pseudopotentials and an exchange correlation functional in the form of a Perdew–Burke–Ernzerhof functional with the van der Waals interaction by the empirical dispersion correction. Vacuum regions of at least 20 Å in vertical and parallel directions, respectively, were applied to avoid unphysical interactions between periodic images. All calculations were conducted with a plane wave cutoff of 60 Rydberg and a 3 × 3 × 1 Monkhorst–Pack k-grid for the Brillouin zone, which are sufficient to ensure convergence. Geometry optimization was performed using a quasi-Newton algorithm. A total energy convergence of 1.4 × 10^−4^ eV and residual forces below 0.02 eV Å^−1^ were achieved. The most acceptable pathway for OER is a four-electron transfer process, accompanied by the break of O–O bond and the formation of O–H bond.

## Results and Discussion

### Design and Characterization of Ni–Fe@C_1D&@2D_ Porous Network

**Fig. 1:**
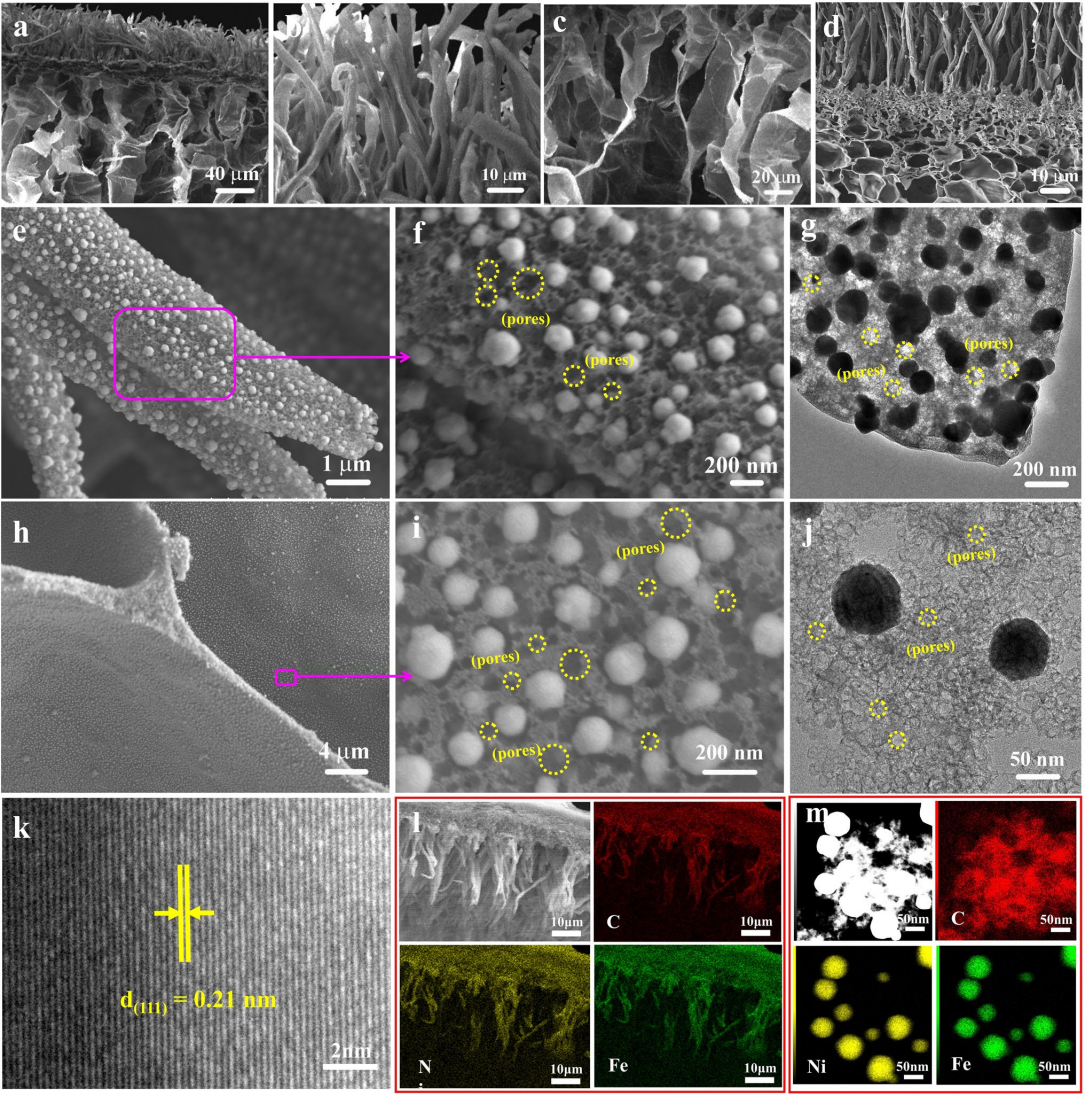
**a**–**c** SEM images of e-fungus; **d**–**f**, **h**–**i** SEM images; **g**, **j** TEM images; **k** HRTEM image; **l**, **m** EDS mappings of Ni–Fe@C_1D&@2D_ porous network

In the experiment the original e-fungus was dried and used directly. Its SEM image (Fig. 1a) reveals an exquisite 3D complex architecture with neat rod-arrays on the top surface (Fig. 1b) and interlaced-sheets network below (Fig. 1c). Small pieces of e-fungus were immersed in Ni(NO_3_)_2_·6H_2_O and Fe(NO_3_)_2_·9H_2_O aqueous solution at room temperature and then dried through a freeze-drying process to obtain metal salts@e-fungus hybrid, which was subsequently pyrolyzed under N_2_ atmosphere to generate black powders. The detailed synthesis process is illustrated in Fig. S1. The resulted black powders were characterized by SEM and TEM. The sample inherits the e-fungus structure with interlaced-sheets network at the bottom and ordered rod-arrays on the top (Fig. 1d). Ni–Fe alloy nanoparticles with an average diameter of ~ 150 nm are homogenously dispersed on both the rod-arrays and the interlaced-sheets (Fig. 1e, f and h, i). TEM images (Fig. 1g, j) further confirm the results from SEM observations. It is worth noting that a huge number of deep nanopores spread on the whole e-fungus-inherited carbon structure, marked with yellow circles shown in SEM images (Fig. 1f, i) and TEM images (Fig. 1g, j), leading to a special multilevel carbon rod-arrays@interlaced-sheets network with numerous open, interconnected and hierarchical pores. XRD pattern (Fig. S2a) reveals that the nanoparticles are typical Ni–Fe alloy (JCPDS card No. 12-0736). Crystal planes with a d-spacing of 0.21 nm in HRTEM image (Fig. 1k), corresponding to (111) lattice of the Ni–Fe alloy, confirm the formation of crystalline Ni–Fe alloy nanoparticles on the 3D bio-carbon structure (Ni–Fe@C_1D&2D_ porous network). Elemental mappings of Ni–Fe@C_1D@2D_ porous network (Fig. 1l) reveal the uniformly dispersion of Ni and Fe within the carbon rod-arrays@interlaced-sheets network. The high-angle annular dark-field scanning TEM (HAADF-STEM) image and the corresponding energy-dispersive spectroscopy (EDS) elemental mappings (Fig. 1m) show that both Ni and Fe atoms uniformly distribute over the nanoparticles, confirming Ni and Fe are well alloyed. XPS spectra (Fig. S2b-e) indicate the existence of metallic Ni and Fe, oxidized species of Ni and Fe from the partial surface oxidization and two C 1*s* peaks corresponding to C–C and C=O [28, 29].

### Structure Promotion and Pore Creation Mechanism

**Fig. 2:**
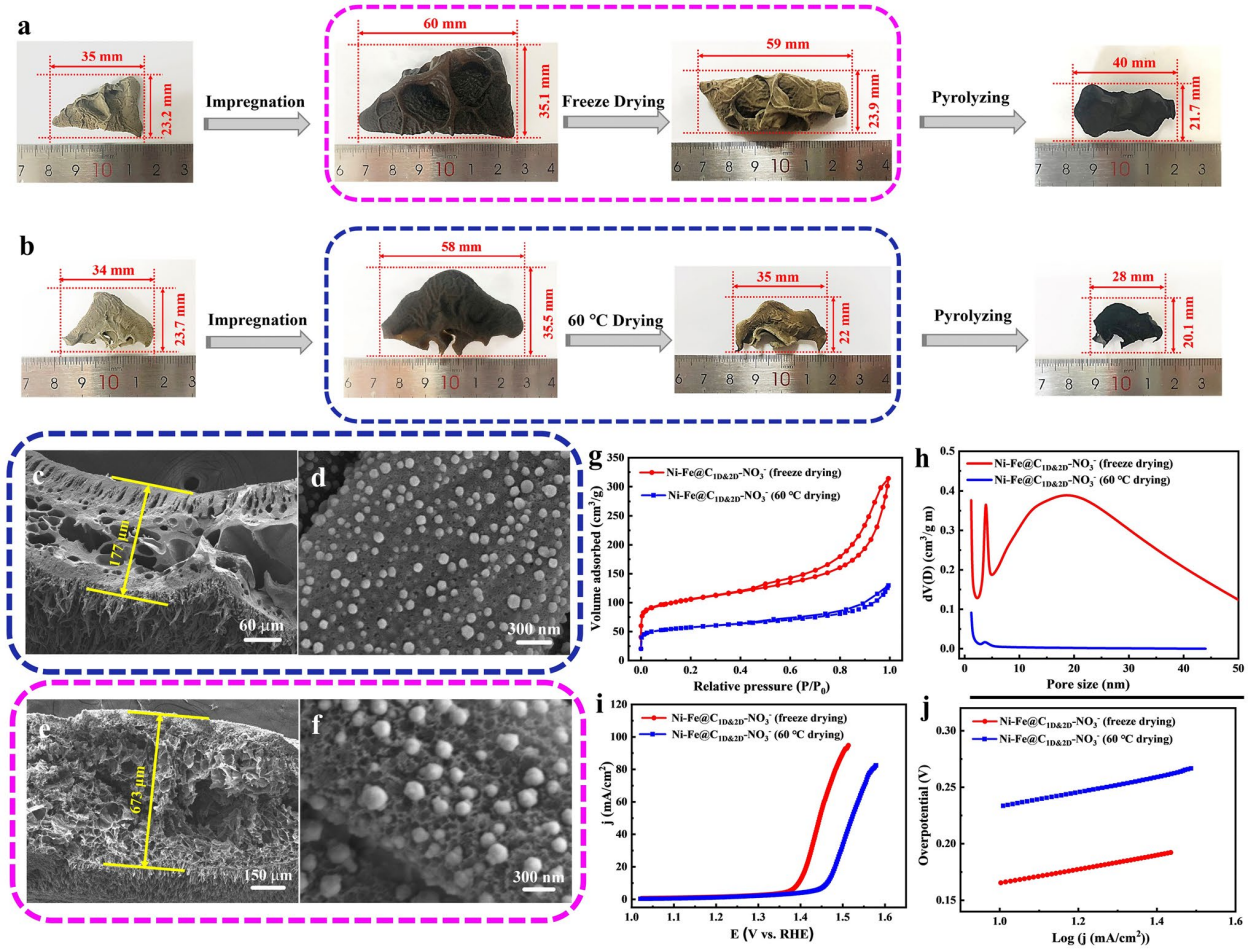
The comparisons between the Ni–Fe@C_1D@2D_-NO_3_^−^ porous network prepared through freeze-drying and oven-drying (60 °C): **a**, **b** The comparative digital photographs at different synthesis stages; **c**, **d** SEM images for oven-drying; **e**, **f** SEM images for freeze-drying; **g** Nitrogen adsorption–desorption isotherms; **h** Pore size distributions; **i** OER polarization curves obtained at a scan rate of 5 mV s^−1^ and **j** Corresponding Tafel plots

During the synthesis process, besides taking advantages of the special structure and characteristics of e-fungus, two technical steps have extraordinary effects on catalyst’s final structure and thus on its superior electrocatalytic activity, one is the drying method and the other is the used metallic reactants. Figure 2a, b show the photos of the e-fungus before and after the impregnation, clearly demonstrating the great volume swelling and the ultra-high water absorbing capacity of e-fungus, resulting in highly active molecules/ions loading. The e-fungus with metal salts was then dried through conventional oven-drying and freeze-drying process, respectively. Figure 2c–f displays their corresponding SEM images. Compared to freeze-drying, oven-drying (60 °C) makes the e-fungus shrink greatly, as shown in Fig. 2c, d that the rods are aggregated and the interlaced-sheets network even can’t be kept after oven-drying (60 °C), while the exquisite 3D complex architecture with neat rod-arrays on the top surface and interlaced-sheets network at the bottom can be perfectly maintained after freeze-drying (Fig. 2e, f). The sample through freeze-drying obviously has a better space geometrical maintain than that through conventional oven-drying. As a result, the sample prepared through freeze-drying gives a much higher specific BET surface area of 358.7 m^2^ g^−1^ and pore volume of 0.490 cm^3^ g^−1^ than that through oven-drying (60 °C) with a BET surface area of only 43.96 m^2^ g^−1^ and a pore volume of 0.02 cm^3^ g^−1^ as shown in Fig. 2g, h. High specific BET surface area and pore volume mean high space geometrical porous structure and high electrode/electrolyte contact area, benefiting OER electrochemical performances (Fig. 2i, j). In a word, freeze-drying after chemical impregnation shows much better effect on the space geometrical maintain than conventional oven-drying, thus greatly increasing its catalytic efficiency.

**Fig. 3:**
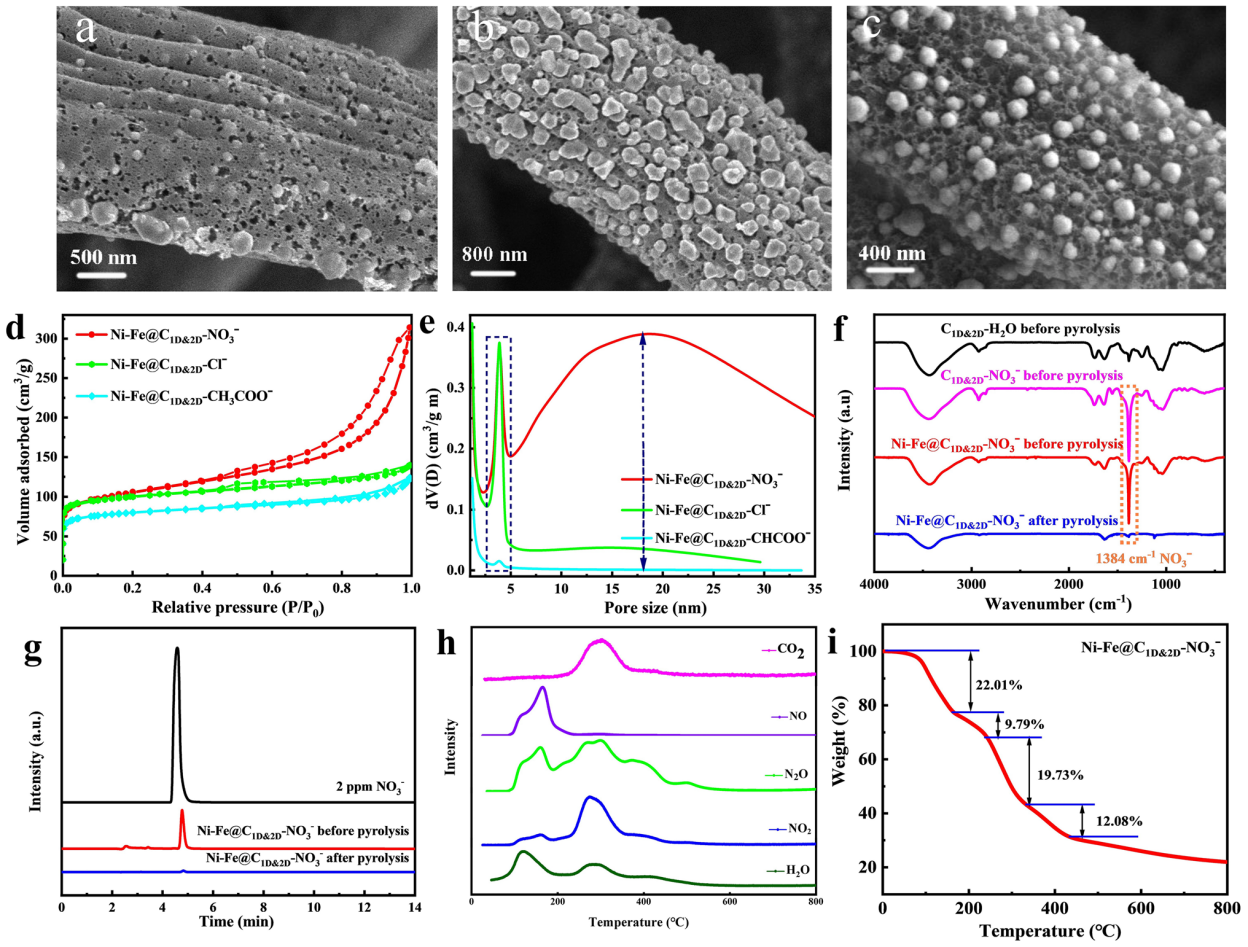
**a**–**c** SEM images; **d** N_2_ adsorption–desorption isotherms and **e** Pore size distributions of Ni–Fe@C_1D@2D_–CH_3_COO^−^, Ni–Fe@C_1D@2D_–Cl^−^ and Ni–Fe@C_1D@2D_–NO_3_^−^; **f** FT-IR spectra of the samples at different stages with NO_3_^−^; **g** NO_3_^−^ ion chromatographic curves of different samples; **h** The decomposition products of Ni–Fe@C_1D@2D_–NO_3_^−^ precursor detected by in situ mass spectrometry upon heating during thermogravimetric analysis; **i** TGA-curve of Ni–Fe@C_1D@2D_–NO_3_^−^ precursor

During the synthesis process, the nitrate treatment process, using nickel nitrate and iron nitrate as metal sources, is important for the formation of Ni–Fe@C_1D@2D_ porous network (also termed as Ni–Fe@C_1D@2D_–NO_3_^−^). In order to find the key point to this special structure, the products synthesized with different salts have been carefully investigated. SEM images in Fig. 3a, b reveal that Ni–Fe@C_1D@2D_–CH_3_COO^−^ has a few non-uniform alloy particles with some small and shallow pores on carbon matrix, while Ni–Fe@C_1D@2D_–Cl^−^ has many alloy nanoparticles on the surface of carbon matrix with some small shallow pores. In contrast, Ni–Fe@C_1D@2D_–NO_3_^−^ consists of spherical Ni–Fe alloy nanoparticles on carbon matrix with open, uniform and deep nanopores on the carbon structure (Fig. 3c), quite different from Ni–Fe@C_1D@2D_–Cl^−^ and Ni–Fe@C_1D@2D_–CH_3_COO^−^. The special porous structure in Ni–Fe@C_1D@2D_–NO_3_^−^ provides a specific BET surface area of 358.7 m^2^ g^−1^, higher than those of Ni–Fe@C_1D@2D_–CH_3_COO^−^ (266.6 m^2^ g^−1^) and Ni–Fe@C_1D@2D_–Cl^−^ (333.8 m^2^ g^−1^) (Fig. 3d and Tab. S1). Meanwhile, as shown in Fig. 3e, Ni–Fe@C_1D@2D_–CH_3_COO^−^, Ni–Fe@C_1D@2D_–Cl^−^ and Ni–Fe@C_1D@2D_–NO_3_^−^ all have small mesopores distributing around 4 nm, which come from the microstructure of e-fungus. Interestingly, Fig. 3e also directly reveals that Ni–Fe@C_1D@2D_–NO_3_^−^ has a wide distribution of pores around 18 nm with very large pore volume, besides the small 4 nm e-fungus-inherited pores. This result is in good agreement with SEM and TEM observations in Fig. 1e–j showing a special porous structure with ~ 18 nm nanopores all over the carbon matrix.

To further confirm the pore-creating mechanism, e-fungus was immersed into pure acids (same anion concentration as the metal salts, same below), HCl, CH_3_COOH and HNO_3_, respectively, to simplify the system environment and avoid the effect of metal ions. The sample immersed in deionized water was used as reference. After the pyrolysis process, the synthesized 3D carbon structures (termed as C_1D@2D_–HCl, C_1D@2D_–CH_3_COOH, C_1D@2D_–HNO_3_ and C_1D@2D_–H_2_O, respectively) have been carefully characterized. SEM images (Fig. S3a–c) reveal that though C_1D@2D_–HCl and C_1D@2D_–CH_3_COOH have a little rougher surface than C_1D@2D_–H_2_O, they all have no obvious pores on carbon matrix. This result indicates that Cl^−^ or CH_3_COO^−^ is not the key factor for porous structure. Obviously, the sample synthesized in HNO_3_ solution, C_1D@2D_–HNO_3_, has a special porous structure with a huge number of pores all over the carbon structure (Fig. S3d), different from Ni–Fe@C_1D@2D_–Cl^−^ and Ni–Fe@C_1D@2D_–CH_3_COO^−^ but quite similar to Ni–Fe@C_1D@2D_–NO_3_^−^. It directly confirms the critical effect of NO_3_^−^ for these deep nanopores all over the carbon matrix. To further investigate the effect of NO_3_^−^ and explain the formation mechanism, FT-IR and IC have also been employed. As shown in Fig. 3f, besides the peaks attributed to the natural e-fungus, there is a worth noting peak at 1384 cm^−1^ assigned to NO_3_^−^ from C_1D@2D_–NO_3_^−^ or Ni–Fe@C_1D@2D_–NO_3_^−^ precursor before pyrolysis (with C_1D@2D_–H_2_O as reference), which becomes weak after pyrolysis. This result confirms the high amount of NO_3_^−^ ion before pyrolysis and the successful decomposition of NO_3_^−^ after pyrolysis, which is in agreement with the result from IC results (Fig. 3g). From the curves of the samples after pyrolysis, it could be seen that the decomposition and carbonization processes are quite complete and no organic compounds remain. As shown in Fig. 3h, composition analysis of evolved gases from TGA supports the decomposition of Ni–Fe@C_1D@2D_–NO_3_^−^ precursor before pyrolysis into gases such as H_2_O, NO_2_, N_2_O, NO and CO_2_, which further confirms that the nitrate can be decomposed to produce a mass of gases. With the decomposition reaction of the Ni–Fe@C_1D@2D_–NO_3_^−^ precursor proceeding, the gas bubbles become more and bigger, leading to the large-scale pore creation. This significant difference would be attributed to the intrinsic properties of the added metal salts and e-fungus, leading to different decomposition/reaction and carbonization/solidification processes. As shown in Fig. S4, acetate ions wouldn’t react with e-fungus but decompose at quiet high temperature (~ 300 °C). E-fungus has similar pyrolysis temperature as acetate ions. The almost simultaneous decomposition of acetate and e-fungus is no doubt unbeneficial for the pore formation and just a few small and shallow pores can be created. Compared with acetates, chlorides are more difficult to be pyrolyzed. With the increase of the reaction temperature, they would react with e-fungus, releasing some gas and leading to the gradual solidification of e-fungus with some small and shallow pores left in carbon matrix. So, when these two kinds of salts decompose, the e-fungus bases are almost or already full-carbonized, difficult to be bent or squeezed much by the gas resulting from the decomposition reaction. Thus, the large-scale pore-creating can’t be realized during the pyrolysis process for Ni–Fe@C_1D@2D_–CH_3_COO^−^ and Ni–Fe@C_1D@2D_–Cl^−^. However, as shown in Fig. 3i, the case of nitrate is quite different. The decomposition of nitrate begins at below 200 °C, which is lower than the beginning temperature of e-fungus carbonization/solidification. It means that when the decomposition of nitrate begins, the carbonization of e-fungus hasn’t begun. With the decomposition reaction proceeding, the gas bubbles become more and bigger and then the e-fungus-base gradually decomposes and solidifies, leading to the large-scale pore creation, something like the food fermentation process.

**Fig. 4:**
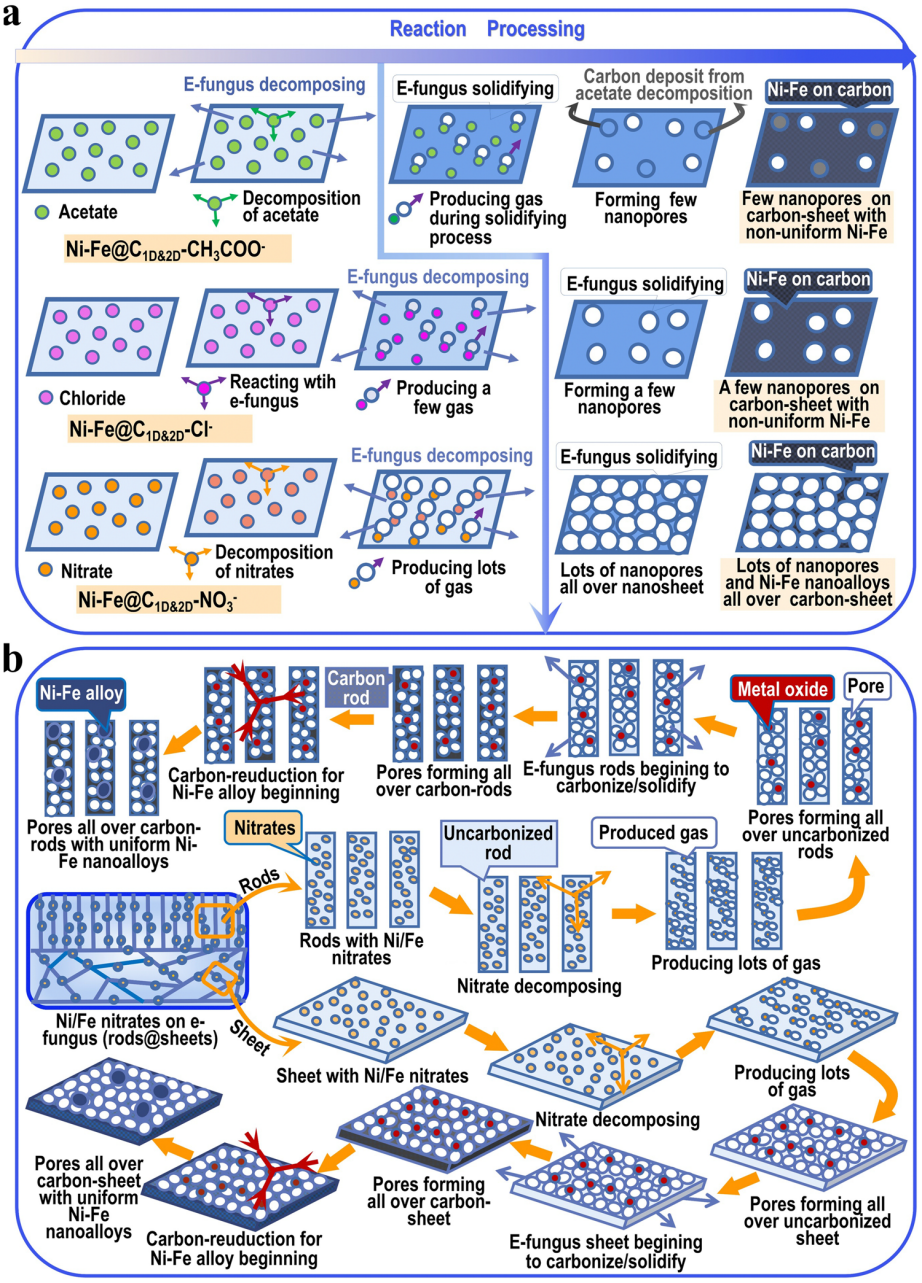
**a** Possible mechanism schemes for Ni–Fe@C_1D&2D_ porous networks with chlorides and acetates as the reactants; **b** Scheme of CF pore-creating mechanism for Ni–Fe@C_1D@2D_–NO_3_^−^

Based on the above discussion, we propose a chemical fermentation (CF) mechanism of the pore forming process as is shown in Fig. 4a, b. Thereinto, the fermentation is broadly known as an enzymatically controlled effervescence transformation and widely used to make loose food, such as bread and pizza. In our case, it is a chemical decomposition-controlled effervescence, followed by the solidification/carbonization process, and the created pores are nanopores, much smaller than the pores produced by conventional fermentation. Moreover, this pore-creating process happens in materials synthesis process and depends on the well matching of two chemical processes—decomposition and solidification. Thus, in our experiment, this brand-new pore-creating mechanism through CF in materials is not only proposed for multilevel complex structure with massive, interconnected and hierarchical channels but also confirmed to be an effective structure-promoting strategy for the achievement of high-performance catalysts. The decomposition reaction equations are shown as follows:

3 $${\text{M}}\left( {{\text{NO}}_{3} } \right)_{n} + \, C_{x} \left( {{\text{H}}_{2} {\text{O}}} \right)_{y} \left( {{\text{biomass}}} \right) \, \, \,\underrightarrow{\text{heating}} \, \, \,{\text{ NO}}_{2} \uparrow + {\text{ N}}_{2} {\text{O}} \uparrow + {\text{ NO}} \uparrow + {\text{ CO}}_{2} \uparrow + {\text{ H}}_{2} {\text{O}} + {\text{M}}_{2} {\text{O}}_{n} \,\,\,\,\,n = 2\,{\text{or}}\,3;{\text{ M}} = {\text{Ni}}\,{\text{or}}\,{\text{Fe}}$$

4 $${\text{Fe}}_{2} {\text{O}}_{3} + {\text{NiO}} + C\,\,\,\underrightarrow{\text{heating}}\,\,{\text{ NiFe}} + {\text{CO}}_{2} \uparrow$$

This structure formed through CF pore-creating mechanism could be triggered by simple synthesis process, such as nitrate treatment, but has an essential requirement, that is, the well matching of gasification and solidification. It proposes an efficient structure-promoting concept and brings up a brand-new strategy to create deep, interconnected and hierarchical pores all over the materials, which is very important for the achievement of high-performance functional materials.

### Electrochemical Evaluations of Ni–Fe@C_1D&@2D_ Porous Network

**Fig. 5:**
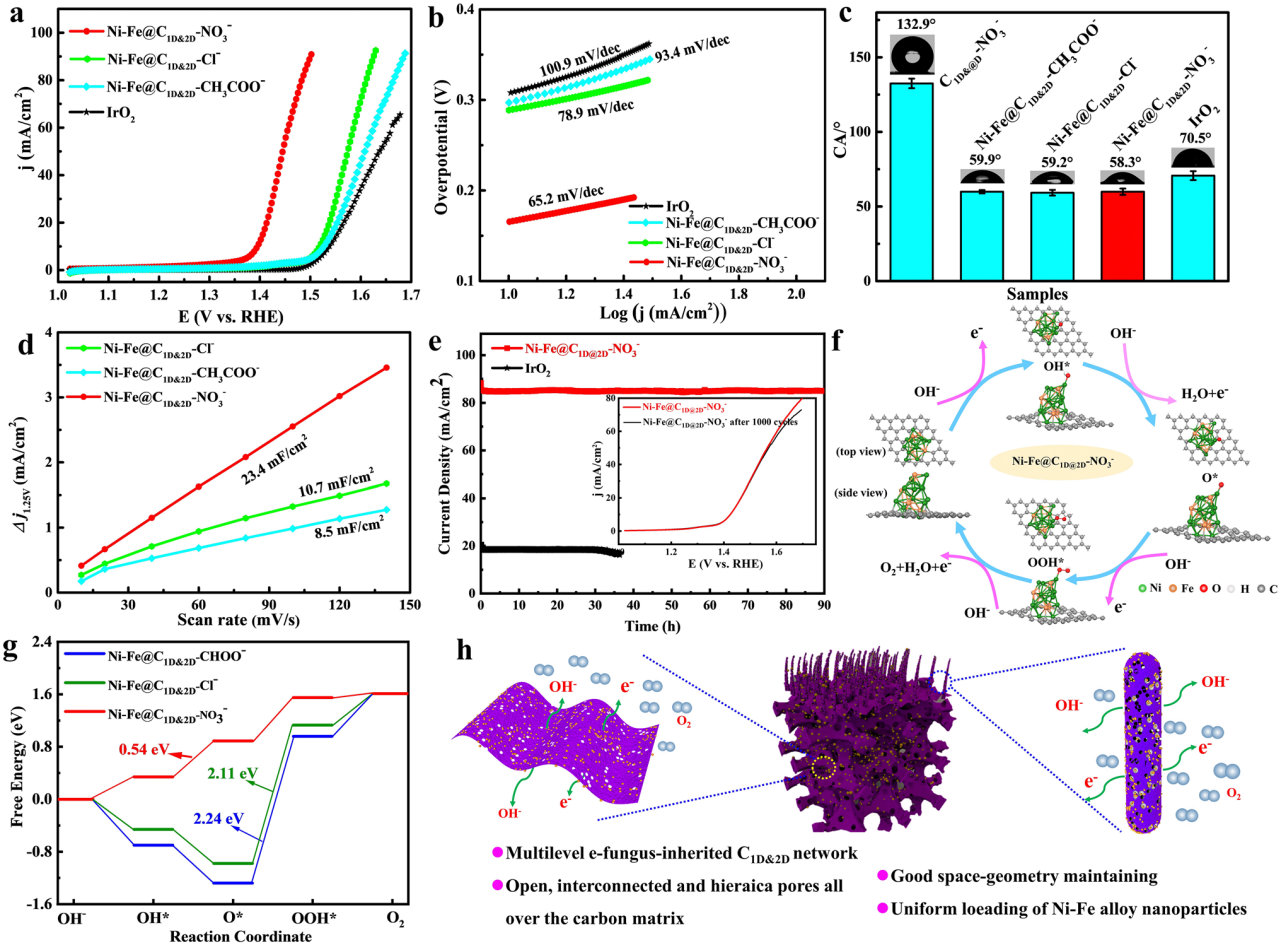
**a** OER polarization curves obtained at a scan rate of 5 mV s^−1^; **b** Corresponding Tafel plots; **c** Contact angles; **d** Corresponding capacitive currents at 1.25 V (*vs.* RHE) as a function of scan rate of the Ni–Fe@C_1D&2D_ porous networks prepared with different salts; **e** Long-term stability test of the Ni–Fe@C_1D&2D_–NO_3_^−^ porous networks and IrO_2_; **f** OER process on Ni–Fe@C_1D&2D_-NO_3_^−^; **g** Gibbs free energy changes (*U* = 0) of each reaction step; **h** Schematic diagram and advantages of the Ni–Fe@C_1D&2D_–NO_3_^−^ porous networks for OER

After the structure characterization in details, the OER electrocatalytic performances of Ni–Fe@C_1D@2D_–CH_3_COO^−^, Ni–Fe@C_1D@2D_–Cl^−^ and Ni–Fe@C_1D@2D_-NO_3_^−^ were carefully evaluated. Ni–Fe@C_1D@2D_–Cl^−^ and Ni–Fe@C_1D@2D_–CH_3_COO^−^ exhibit OER activity with the overpotential of 288 and 297 mV, respectively, to drive a current density of 10 mA cm^−2^, while commercial IrO_2_ needs 308 mV (Fig. 5a). With the special hierarchical pore structures from CF pore creation, Ni–Fe@C_1D@2D_–NO_3_^−^ possesses much better OER catalytic activity, only requiring an ultralow overpotential of 165 and 270 mV at 10 and 40 mA cm^−2^ on non-supported inert electrode, respectively. Its better OER activity could also be revealed by the Tafel slope. As shown in Fig. 5b, the Tafel slope of Ni–Fe@C_1D@2D_–NO_3_^−^ (65.2 mV dec^−1^) is much smaller than those of Ni–Fe@C_1D@2D_-Cl^−^ (78.9 mV dec^−1^), Ni–Fe@C_1D@2D_-CH_3_COO^−^ (93.4 mV dec^−1^) and IrO_2_ (100.9 mV dec^−1^), which indicates that it has the fastest reaction kinetics. The hydrophilic surface of Ni–Fe@C_1D@2D_–NO_3_^−^ has been confirmed by its smallest water contact angle between water and corresponding electrode (58.3°) (Fig. 5c), indicating best electrolyte wettability of the catalyst. The easy absorption of the electrolyte into the catalyst and the well spreading on the porous walls of the rod-arrays@interlaced-sheets network to form a continuous OH^−^ ion transport layer benefit the catalytic performance. According to the solid–liquid–gas interface theory, structures with roughness at both micro- and nanoscale can reduce the contact region between the bubbles and electrode, leading to a low interfacial adhesion of bubbles and facilitating the wettability toward the electrolyte [30]. Such rough hydrophilic surface mainly comes from the porous structure on C_1D@2D_ network formed through CF pore-creating mechanism. In other words, the open, deep and numerous nanopores on C_1D@2D_ network not only expose a great number of active sites but also facilitate the diffusion of the electrolyte, which helps the improvement of electrocatalytic performances.

To further demonstrate the superior catalytic performance, Nyquist plots (Fig. S5) of electrochemical impedance spectroscopy (EIS) reveal that Ni–Fe@C_1D@2D_–NO_3_^−^ exhibits the smallest impedance arc radius among all samples. This phenomenon indicates significantly enhanced charge-transfer kinetics at the catalyst–electrolyte interface, which is attributed to the synergistic interplay between the hierarchical 3D porous carbon architecture and the homogeneously distributed Ni–Fe alloy nanoparticles [31, 32]. Electrochemical active surface areas (ECSA), reflected by double-layer capacitance (C_dl_), were also calculated to investigate the electrode materials’ intrinsic activities. Measured through CV method and shown in Fig. S6 and 5d, the C_dl_ of Ni–Fe@C_1D@2D_–NO_3_^−^ is 11.7 mF cm^−2^ at 1.25 V, much higher than those of Ni–Fe@C_1D@2D_-CH_3_COO^−^ (4.25 mF cm^−2^) and Ni–Fe@C_1D@2D_–Cl^−^ (5.35 mF cm^−2^), further confirming Ni–Fe@C_1D@2D_–NO_3_^−^ a much better electrocatalyst for OER than the others. As shown in Fig. 5e, Ni–Fe@C_1D@2D_ porous network also displays excellent durability with only a slight decay (less than 3%) of high current density 86 mA cm^−2^ after 90 h, while the IrO_2_ only remains 68.3% at current density of 18 mA cm^−2^ for 37 h, revealing a great long-term stability of Ni–Fe@C_1D@2D_ porous network, which is also an important criterion to assess electrocatalysts in practical application. The Ni–Fe@C_1D@2D_–NO_3_^−^ catalysts after OER 1000 cycles were characterized by XRD, TEM, HRTEM and XPS, and the corresponding results are presented in Fig. S7. TEM images in Fig. S7a, b confirm that the nanoparticles with an average diameter of ~ 150 nm are homogenously dispersed on both rod-arrays and interlaced-sheets after OER 1000 cycles test, which possess the similar structure to the original catalyst. Compared with the Ni–Fe@C_1D@2D_–NO_3_^−^ before OER (Fig. S7c), the surface changes partly after OER 1000 cycles. Figure S7d shows an HRTEM image of Ni–Fe@C_1D@2D_–NO_3_^−^ nanoparticles after an alkaline OER catalytic process, and an amorphous NiFe oxo-/hydroxide layer of 4 nm is observed. As shown in Fig. S7e, the XRD pattern reveals that the nanoparticles are typical Ni–Fe alloy (JCPDS-12-0736). However, the intensity is very weak and there is a broad peak around 22°, which may be attributed to the formation of the amorphous NiFe oxo-/hydroxide layer after the OER catalytic process. XPS spectra (Fig. S7f-g) indicate the existence of metallic Ni and Fe consistent with XRD and HRTEM results, and oxidized species of Ni and Fe from the partial oxidization during the OER catalytic process. In a word, after 1000 cycles, although amorphous hydroxide is produced on the surface of the alloy particles, the overall structure and composition are still maintained, which further confirms the long-term stability of Ni–Fe@C_1D@2D_-NO_3_^−^ porous network. Furthermore, Fig. S8 presents a multistep chronopotentiometric curve for Ni–Fe@C_1D@2D_ porous network with the anodic current density increasing from 10 to 100 mA cm^−2^ with an increment of 10 mA cm^−2^. The potential immediately levels off at 1.401 V for the start current value and remains constant for the rest 500 s. Moreover, all other steps also show similar results, demonstrating the good conductivity, mass transportation and mechanical robustness of Ni–Fe@C_1D@2D_ porous network electrode. As shown in Fig. S9, by applying a constant disk current (300 µA), O_2_ molecules generated on the surface of Ni–Fe@C_1D@2D_ porous network is swept across the surrounding Pt ring electrode and then reduced. A ring current of about 76.42 µA can be obtained, corresponding to a high Faradaic efficiency of 99.5% [33]. Furthermore, Ni–Fe@C_1D@2D_ porous network also has the very low overpotential of 270 mV at high current density of 40 mA cm^−2^. Its catalytic activity has been compared with many excellent OER electrocatalysts reported so far (Fig. S10 and the corresponding references) and the details of parameter comparison are shown in Tab. S2. The reason is that the obtained e-fungus-inherited carbon matrix is a great 3D current collector for fast electron transport. The uniformly dispersed Ni–Fe alloy nanoparticles loading on such anisotropic carbon structure offers more surface area for active molecules and/or ions to anchor and disperse. Moreover, the open, interconnected, hierarchical pores all over the catalyst could easily trap electrolyte to form continuous ion pathways, shorten the diffusion distance, accommodate various products and speed up the catalytic reactions.

As reported, forming multi-metal catalysts through the incorporation of a secondary transition metal will bring much greater catalytic activity for OER compared to a single component counterpart. Being a 3D transition metal, Ni has two unpaired electrons in d-orbital with electronic configuration of 3*d*^8^4*s*^2^. According to the theory of d-band center, the unpaired d orbit valence electrons are always served as the catalytic activity center and interact with s or d electrons of the reactant molecule during chemical reactions [34]. It has been reported that alloying Ni metal with other metals will modify the electronic structure of Ni and leads to a synergistic effect between alloy metals [35]. In order to reveal the incorporation content on the OER performance, Ni–Fe@C_1D@2D_–NO_3_^−^ catalysts with various Ni/Fe ratios were prepared under the same reaction conditions by using the starting materials with different Ni/Fe molar ratios including 1:0, 6:1, 3:1, 1:1 1:3, 1:6 and 0:1. The formed Ni–Fe alloy nanoparticles on carbon matrix with different Ni/Fe molar ratios, confirmed by XRD analyses (Fig. S11a), have also inherited the hierarchical structure of e-fungus. Particularly, the diffraction peaks shift to lower 2θ angles with the increase of iron contents, which is attributed to the large radius of Fe^3+^ as compared to Ni^2+^ and reveals the successful doping of Fe into the Ni lattice. When the obtained samples were used as OER electrocatalyst, with the decrease of Ni content at the Ni/Fe molar ratio from 1:0 to 0:1, the overpotential first decreases and then increases and the sample synthesized with Ni/Fe = 3:1, namely Ni–Fe@C_1D@2D_ porous network mentioned above, exhibits the lowest overpotential of 165 mV at 10 mA cm^−2^ (Fig. S11b, c). According to the Tafel plots (Fig. S11d), different Ni/Fe molar ratios lead to different absorption capacities and reaction rates and Ni–Fe@C_1D@2D_ porous network (Ni/Fe = 3:1) also has the lowest Tafel slope, demonstrating the fastest OER kinetics and superior OER catalytic activity [36, 37]. This is because that when the atom ratio of Ni and Fe is 3:1, the added Fe modifies the electronic structure of Ni to be the best catalytic state. Meanwhile, the NiFe alloy nanoparticles could disperse uniformly with a small-sized particles.

In order to more clearly study the reaction mechanism for the excellent catalytic performances of the porous structure of carbon-loaded NiFe alloy, we performed in situ Raman spectra and detailed DFT calculations. Figure S12a illustrates the in situ Raman spectra of Ni–Fe@C_1D&2D_–NO_3_^−^ during OER. Upon exceeding an applied potential of 0.45 V, distinct nickel–oxygen vibrational modes emerged in the Raman spectra, exhibiting dual peaks at 516 cm^−1^ (e_g_ symmetry) and 526 cm^−1^ (A_g_ symmetry). Concurrently, a characteristic peak at 552 cm^−1^ developed, corresponding to the formation of NiOOH species with mixed Ni^3+^ valence states. This spectral evolution directly correlates with the preferential adsorption of hydroxyl intermediates (OH_ad_) at activated Ni–O sites, initiating the critical OOH_ad_–Ni^3+^ intermediate phase. Additionally, a peak around 680 cm^−1^, assigned to FeOOH, suggested concurrent OH_ad_ on Fe sites. With increasing applied potentials, the Raman spectral sign of FeOOH progressively attenuated, while the NiOOH vibration mode intensified, demonstrating a voltage-dependent transition of active sites from Fe^3+^- to Ni^3+^-dominated species. This potential-induced phase evolution originates from the marked Lewis acidity of Fe^3+^ at lower potentials, which promotes selective hydroxyl adsorption through strong Fe^3+^–OH^−^ orbital interactions. At elevated potentials, Fe^3+^ mediates a thermodynamically driven oxidation state evolution in Ni centers, effectively lowering the Ni^2+^/Ni^3+^ redox activation barrier and enhancing interfacial hydroxyl affinity. These synergistic interactions within the Ni–Fe@C_1D&2D_–NO_3_^−^ architecture facilitate optimized OH^−^ adsorption/activation kinetics at the critical OER potential-determining step, accounting for the observed catalytic enhancement [38]. The potential-dependent active site transition mechanism is schematically illustrated in Fig. S12b, outlining these mechanistic insights. As shown in Fig. S13, three kinds of carbon sheets with different defect sizes were designed first and then Ni-Fe nanoparticles were loaded on these kinds of carbon sheets as calculation models. C_1D@2D_–CH_3_COO^−^ can be described as the carbon with few nanopores (Fig. S13a); C_1D@2D_–Cl^−^ with a few nanopores (Fig. S13b), C_1D@2D_–NO_3_^−^ with lots of nanopores (Fig. S13c) and NiFe alloy (Fig. S13d). After loaded with NiFe alloy nanoparticles, they represent Ni–Fe@C_1D@2D_–CH_3_COO^−^, Ni–Fe@C_1D@2D_–Cl^−^ and Ni–Fe@C_1D@2D_–NO_3_^−^, respectively. Firstly, the three load models are optimized; it shows that Ni–Fe@C_1D@2D_–CH_3_COO^−^ without any hole defects, after loading the NiFe, does not happen too much deformation (Fig. S14), while Ni–Fe@C_1D@2D_–Cl^−^ occurs partially deformation due to the nanopores (Fig. S15). Notably, Ni–Fe@C_1D@2D_–NO_3_^−^ exhibits pronounced structural deformation (Fig. 5f) attributable to its higher nanopore density in the carbon matrix. This morphological evolution indicates an increase in Ni/Fe binding site density compared to Ni–Fe@C_1D@2D_–Cl^−^ and Ni–Fe@C_1D@2D_–CH_3_COO^−^. These findings confirm that the hierarchical porosity actively enhances metal-support interactions through both physical anchoring and electronic effects, synergistically improving charge/mass transfer dynamics critical for catalytic performance [39]. Then the free energy changes of the three models for OER process were calculated. By analyzing the adsorption configuration and adsorption energy of the intermediates, it is found that the adsorption site of O* on the first two models is a bridge site, which has a very strong adsorption of O*, making it very difficult to form OOH*. Thus, the formation of OOH* is the rate-determining step for the Ni–Fe@C_1D@2D_–CH_3_COO^−^ and Ni–Fe@C_1D@2D_–Cl^−^. The free energy of Ni–Fe@C_1D@2D_–CH_3_COO^−^ and Ni–Fe@C_1D@2D_–Cl^−^ goes up to 2.64 and 2.51 eV, which corresponds to high overpotentials (Fig. 5g). However, in the Ni–Fe@C_1D@2D_-NO_3_^−^ due to the lots of pores, the adsorption site of O* is the top site, which has a weak adsorption for O*, thus making it very easy to form OOH*. At this time, OH* changing to O* becomes the rate-determining step, and the corresponding free energy is only 0.54 eV to possess the lowest overpotential. Thus, the superior OER performance can be primarily attributed to the NiFe alloy components. Notably, the OER catalytic activity varies significantly depending on the synthesis conditions of the NiFe alloy. DFT calculations confirm that the transformation of OH* to O* constitutes the rate-determining step in Ni–Fe@C_1D@2D_–NO_3_^−^, a phenomenon amplified by its porous structure. Furthermore, in situ Raman spectroscopy reveals the potential-dependent formation of NiOOH and FeOOH in Ni–Fe@C_1D@2D_–NO_3_^−^, as corroborated by post-cycle HRTEM analysis. These dynamically evolving species optimize hydroxyl ion (OH⁻) adsorption/activation kinetics, thereby accelerating the rate-determining step.

Though the definite effect factors on catalytic performance are still unknown, Ni–Fe@C_1D@2D_ porous network doubtlessly stimulates a remarkable increase of OER activity compared with other catalysts and reaches a very low overpotential on non-substrate inert electrode. The high OER activities of Ni–Fe@C_1D@2D_ porous network can be associated with these reasons (Fig. 5h): (i) A novel multilevel carbon rod-arrays@interlaced-sheets network, inheriting quite well from e-fungus due to its large hydrophilic surface area, strong water absorption and good water retention, enhances the catalyst’s electric conductivity greatly. (ii) The better maintain of space geometrical structure due to the freeze-drying process retains the advantages coming from the e-fungus structure. (iii) A huge number of nanopores all over the carbon matrix created through a brand-new CF pore-creating mechanism provide large, interconnected and free-flowing channels. (iv) The synergistic effect of e-fungus structure, freezing-drying and CF pore creation leads to a special 3D network with numerous open, interconnected and hierarchical pores, which accelerates gaseous exchange and electrolyte permeation, prevents the electrode choking and thus keeps the catalytic reaction go smoothly. (v) The monodispersed Ni–Fe alloy nanoparticles in situ and uniformly embedded in multilevel carbon matrix offer more surface area and active sites.

## Conclusions

In summary, a simple and general biomass-promoted technique has been firstly proposed for a carbon rod-arrays@interlaced-sheets network with open, interconnected and hierarchical pores throughout and homo-dispersive Ni–Fe alloy nanoparticles loading (Ni–Fe@C_1D@2D_ porous network). E-fungus-inherited C_1D@2D_ network increases its electric conductivity and freeze-drying helps space geometrical maintaining. Moreover, an innovative in situ CF pore-creating strategy has been firstly proposed rooting in the well matching of gasification and solidification, leading to a special multilevel carbon network full of open, interconnected and hierarchical pores, which makes the electrolyte facilely permeate into electrocatalyst and offers more surface area and active sites for active molecules or ions to anchor and disperse. As a result, Ni–Fe@C_1D@2D_ porous network exhibits an unprecedented high electrocatalytic activity for OER with an ultralow overpotential of 165 mV at 10 mA cm^−2^ vs RHE, which is the lowest overpotential so far on non-supported inert electrode. Its high catalytic activity, together with a long-term stability of more than 90-h testing with negligible decreasing, far surpasses the performance of current OER electrocatalysts reported to date. Theoretical calculations indicate that the porous structure of the carbon matrix would significantly enhance the interaction between alloy nanoparticles and carbon matrix to promote the electrocatalytic activity and stability. This work not only brings up a brand-new CF pore-creating mechanism for high-efficient electrocatalyst constructing, but also proposes a simultaneous structure-promoting strategy for functionalized multilevel nanostructures designing.

## Supplementary Information

Below is the link to the electronic supplementary material.

Supplementary file1 (DOC 38565 KB)
