# Supplementary material for: Chemical Fermentation PoreCreation on Multilevel Bio-Carbon Structure with In Situ Ni–Fe Alloy Loading for Superior Oxygen Evolution Reaction Electrocatalysis
Source: Nanomicro Lett. 2025 May 21;17:269. doi: 10.1007/s40820-025-01777-2 (PMC12095123; doi:10.1007/s40820-025-01777-2)
Supplement: Supplementary file 1 — Supplementary file1 (DOC 38565 KB) [file 40820_2025_1777_MOESM1_ESM.doc]

**Chemical Fermentation Pore Creation on Multilevel Bio-Carbon Structure with In Situ Ni-Fe Alloy Loading for Superior Oxygen Evolution Reaction Electrocatalysis**

# Qiaoling Kang,^1,3^ Mengfei Su,^1^ Yana Luo,^2^ Ting Wang,^2^ Feng Gao,*^2^ Qingyi Lu*^1^ ^1^ State Key Laboratory of Coordination Chemistry, Coordination Chemistry Institute, Collaborative Innovation Center of Advanced Microstructures, School of Chemistry and Chemical Engineering, Nanjing University, Nanjing 210023, P. R. China.

# ^2^ Department of Materials Science and Engineering, Jiangsu Key Laboratory of Artificial Functional Materials, Collaborative Innovation Center of Advanced Microstructures, College of Engineering and Applied Sciences, Nanjing University, Nanjing 210093, P. R. China.

^3^ College of Materials and Chemistry, China Jiliang University, Hangzhou, 310018, China.

*Corresponding author. E-mail: qylu@nju.edu.cn; fgao@nju.edu.cn


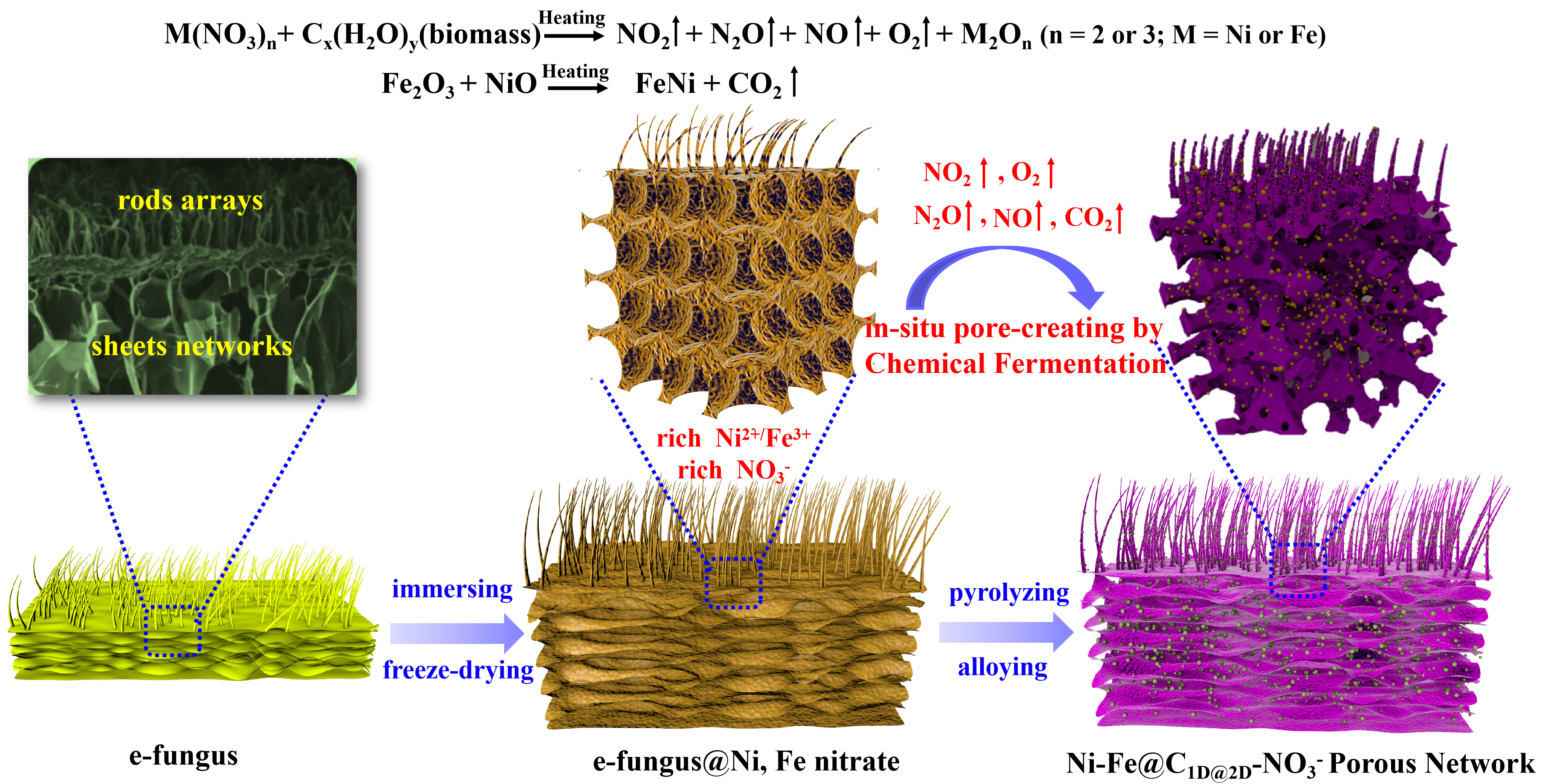


**Fig. S1** The synthesis scheme of Ni-Fe@C_1D&2D_-NO_3_^-^ porous network from e-fungus through in-situ pore creating by Chemical Fermentation.


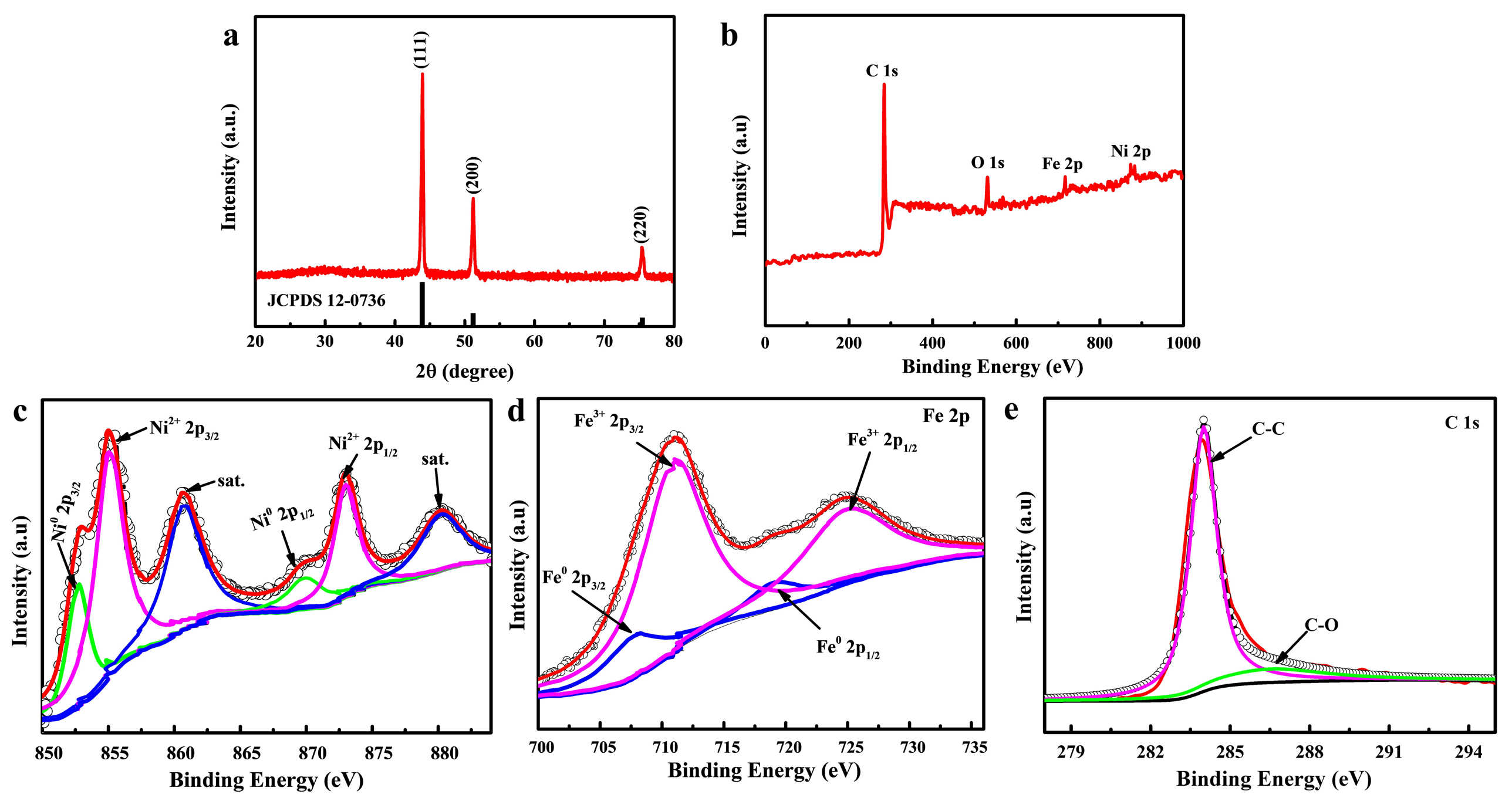


**Fig. S2** (a) XRD pattern and (b~e) Survey and high resolution XPS spectra of Ni-Fe@C_1D&2D_-NO_3_^-^ porous networks.


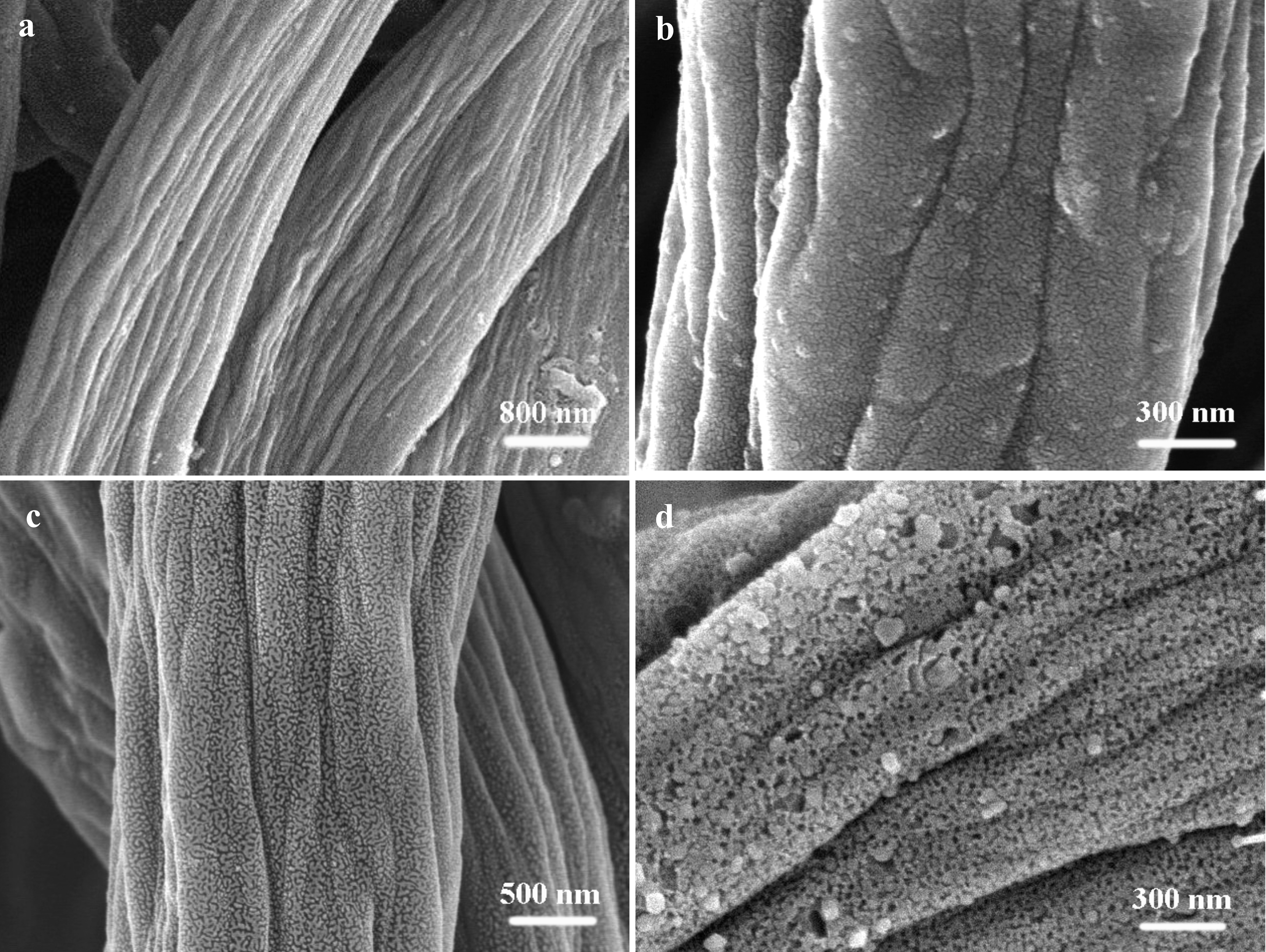


**Fig. S3** SEM images of (a) C_1D&2D_-H_2_O; (b) C_1D&2D_-HCl; (c) C_1D&2D_-CH_3_COOH and (d) C_1D&2D_-HNO_3_.


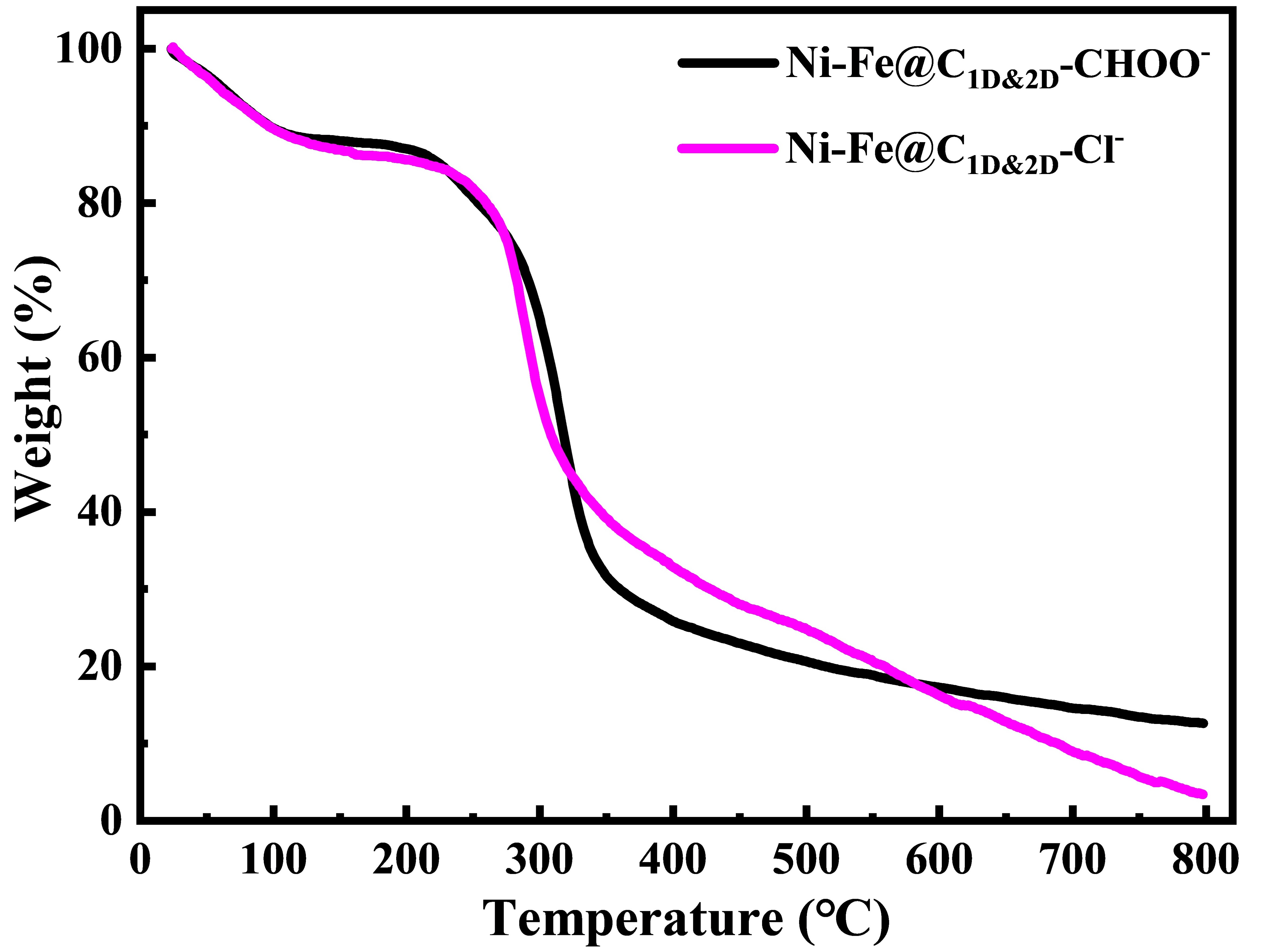


**Fig. S4** TGA-curve of Ni-Fe@C_1D@2D_-CH_3_COO^-^ and Ni-Fe@C_1D@2D_-Cl^-^ precursor.


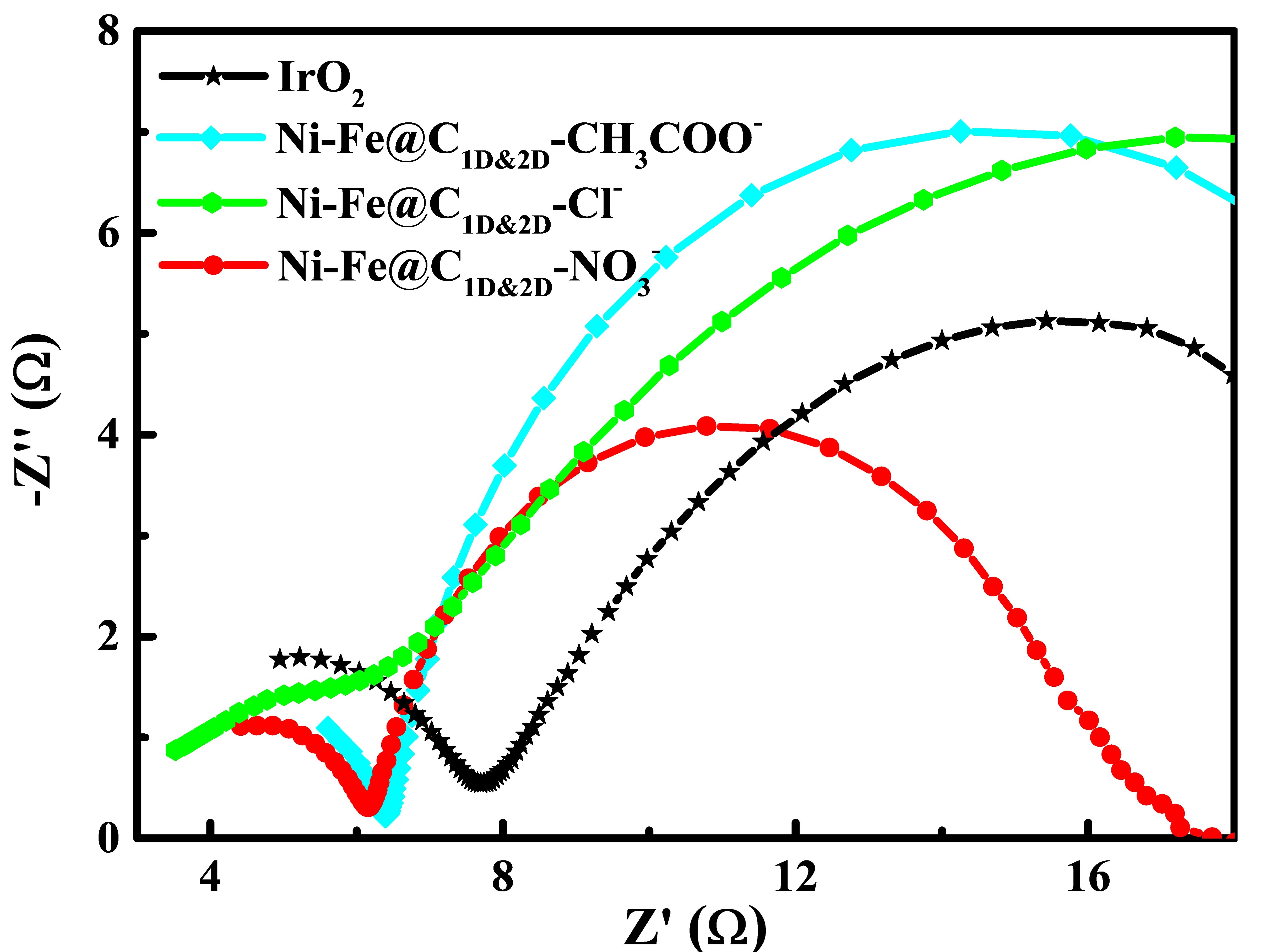


**Fig. S5** Electrochemical impedance spectra of different electrocatalysts.


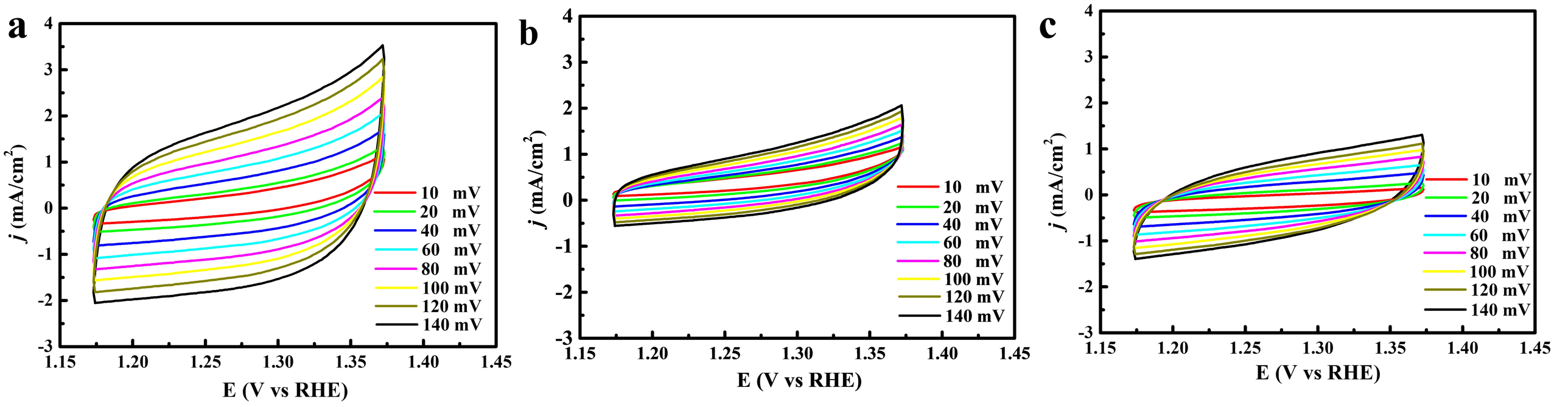


**Fig. S6** CV curves of (a) Ni-Fe@C_1D&2D_-NO_3_^-^, (b) Ni-Fe@C_1D&2D_-Cl^-^ and (c) Ni-Fe@C_1D&2D_-CH_3_COO^-^ porous networks.


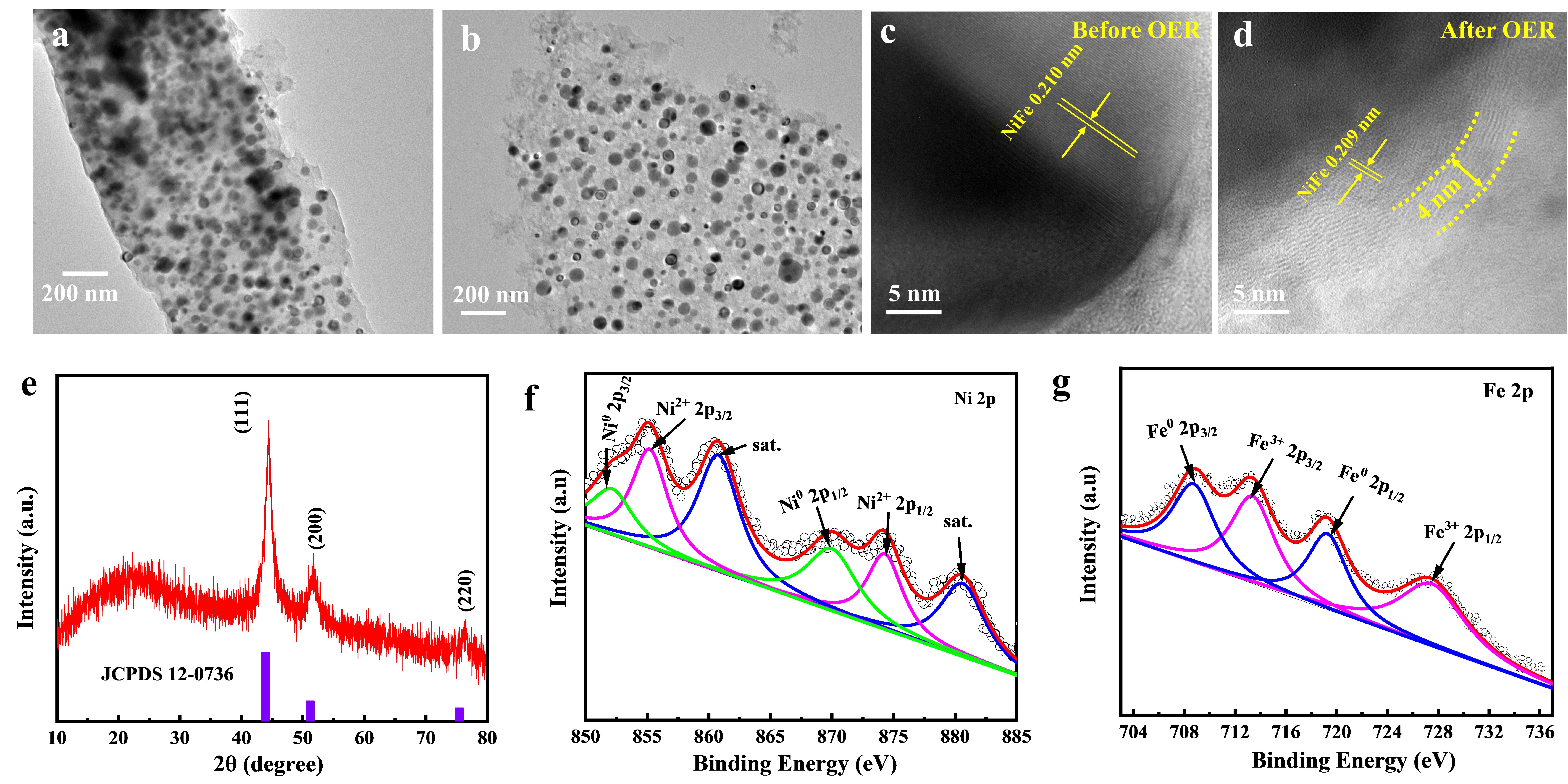


**Fig. S7** (a, b) TEM images of Ni-Fe@C_1D&2D_-NO_3_^-^ after OER 1000 cycles; (c) HRTEM image of Ni-Fe@C_1D&2D_-NO_3_^-^ before OER; (d) HRTEM image of Ni-Fe@C_1D&2D_-NO_3_^-^ after OER 1000 cycles; (e) XRD pattern of Ni-Fe@C_1D&2D_-NO_3_^-^ after OER 1000 cycles; (f, g) High resolution XPS spectra of Ni 2p and Fe 2p of Ni-Fe@C_1D&2D_-NO_3_^-^ after OER 1000 cycles.


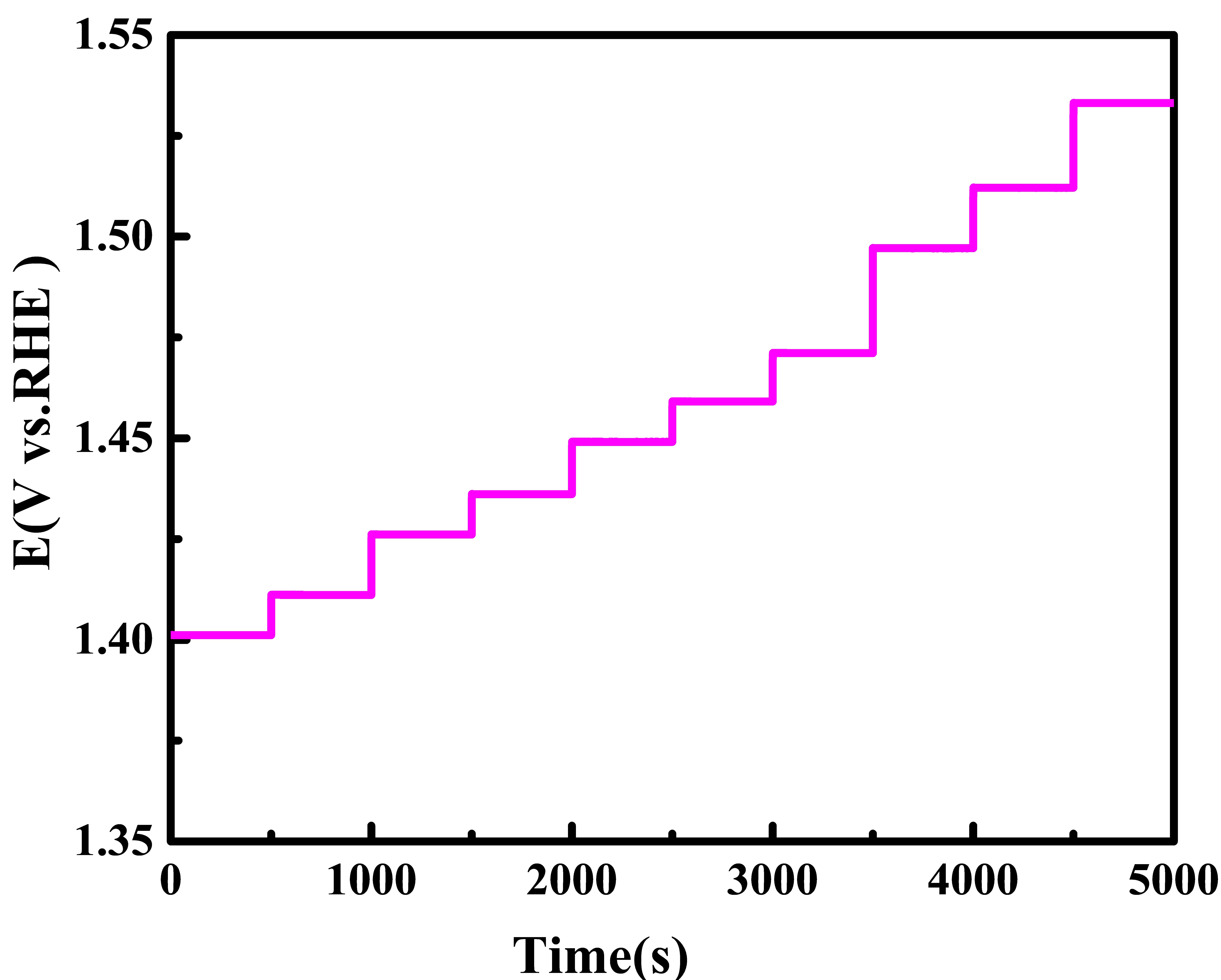


**Fig. S8** Multicurrent process of Ni-Fe@C_1D@2D_-NO_3_^-^ with the current density changing from 10 mA/cm^2^ to 100 mA/cm^2^ with an increment of 10 mA/cm^2^ per 500 s without iR correction.


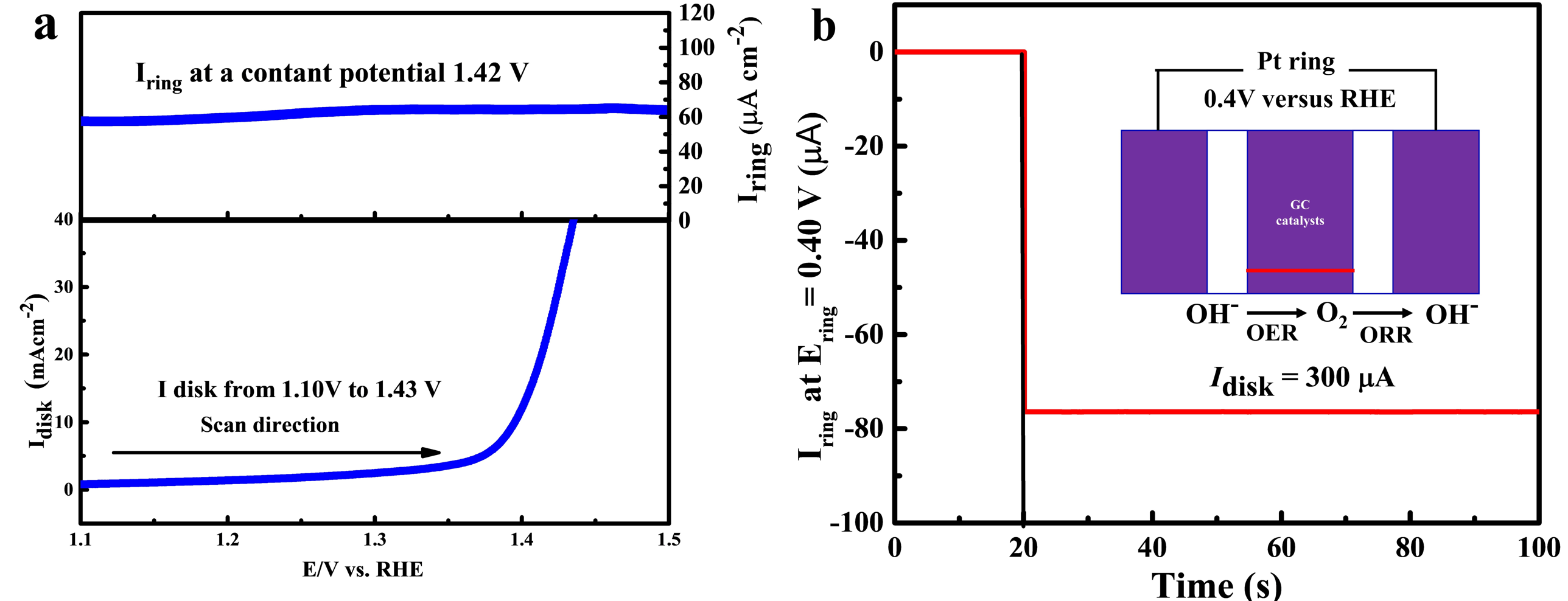


**Fig. S9** (a) RRDE measurement of Ni-Fe@C_1D@2D_-NO_3_^-^ in O_2_-saturated 1 M KOH solution at a rotation speed of 1600 rpm with the ring potential fixed at 1.50 V vs RHE; (b) Faraday efficiency test of Ni-Fe@C_1D@2D_-NO_3_^-^ using the RRDE technique in N_2_-saturated 1 M KOH solution. The inset presents the Faraday efficiency testing mechanism of the RRDE. The blue columns on the both sides are Pt ring electrodes. The Pt ring electrode and glassy-carbon electrode are separated by a non-conductive PTFE barrier (white columns). When a constant current of 300 µA is applied to the disk electrode for O_2_ generation at 20 s, a ring current of ~76.42 µA caused by O_2_ reduction is detected immediately.

Rotating ring-disk electrode (RRDE) technique was employed to investigate the reaction mechanism. The potential of Pt ring electrode was fixed at 1.5 V to oxidize the peroxide intermediates formed at the Ni-Fe@C_1D@2D_-NO_3_^-^ surface during OER. As shown in Figure S3a, compared with the disk current at the scale of mA/cm^2^, only a negligible current density of 63.8 μA/cm^2^ was detected on the ring electrode, indicating negligible formation of hydrogen peroxide and thus a desirable four-electron pathway for water oxidation (4OH^-^ → O_2_ + 2H_2_O + 4e^-^). Meanwhile, a continuous OER (disk)-oxygen reduction reaction (ORR) (ring) process was carried out to verify that the detected disk current was resulted from oxygen evolution rather than other side reactions. By applying a constant disk current (300 µA), O_2_ molecules generated on the surface of Ni-Fe@C_1D@2D_-NO_3_^-^ hybrid catalyst was swept across the surrounding Pt ring electrode and then reduced. A ring current of about 76.42 µA can be obtained, corresponding to a high Faradaic efficiency of 99.5%.


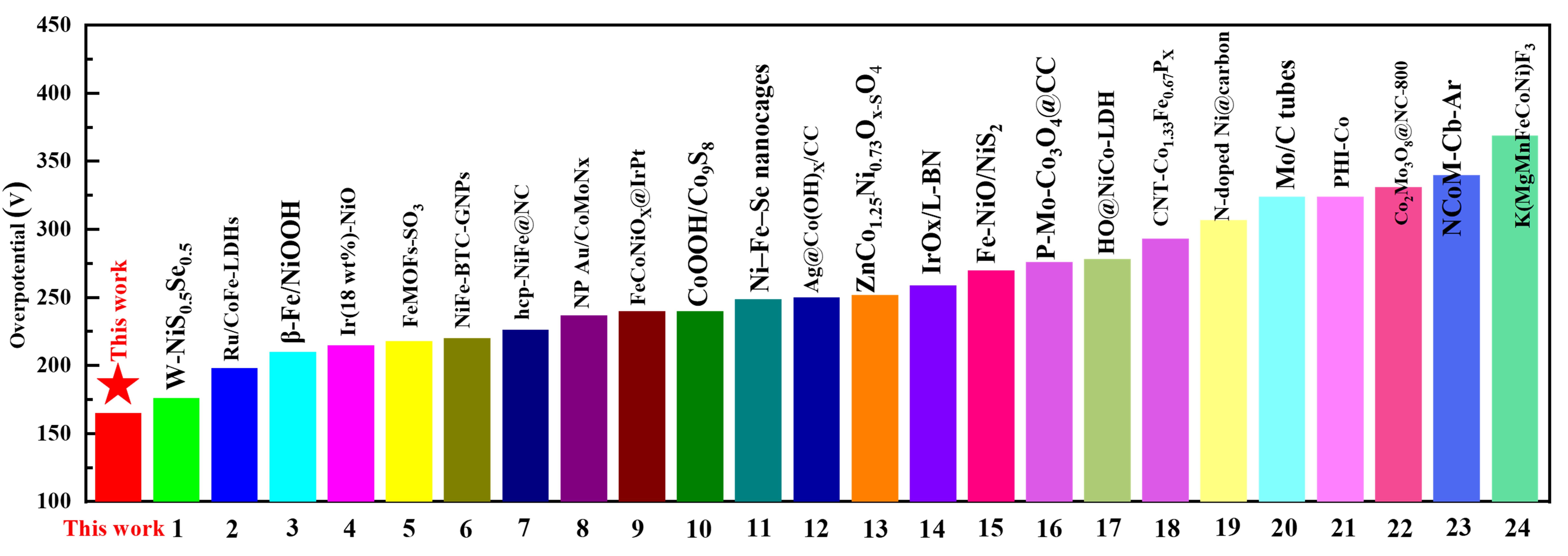


**Fig. S10** Overpotential comparisons at the current of 10 mA/cm^2^ between the Ni-Fe@C_1D&2D_-NO_3_^-^ porous networks and recently reported non-noble metallic electrocatalysts. The corresponding references are listed in Tab. S2.


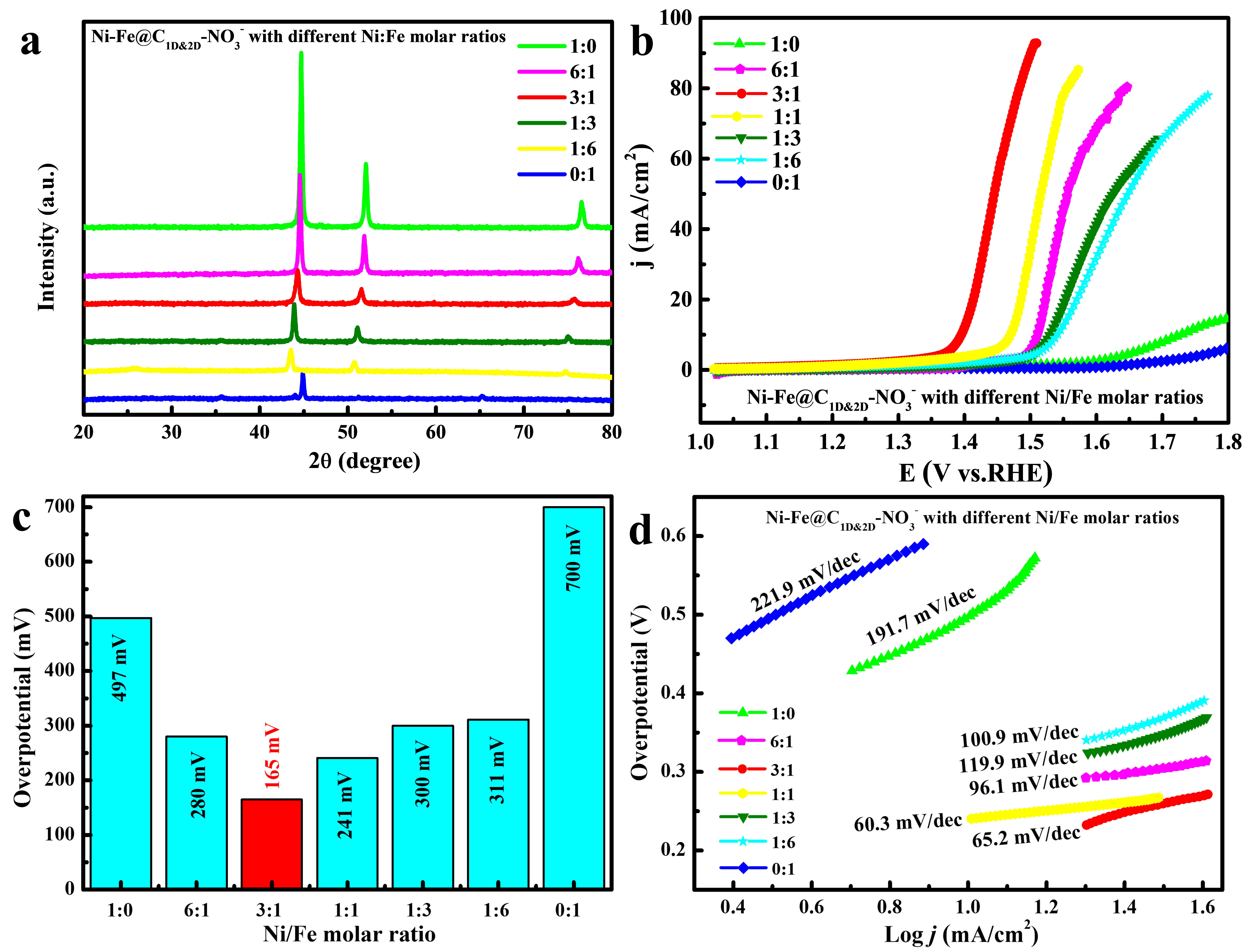


**Fig. S11** (a) XRD patterns; (b) OER polarization curves; (c) Overpotential and (d) Corresponding Tafel plots of the different catalysts with different Ni/Fe molar ratios in 1M KOH solution.


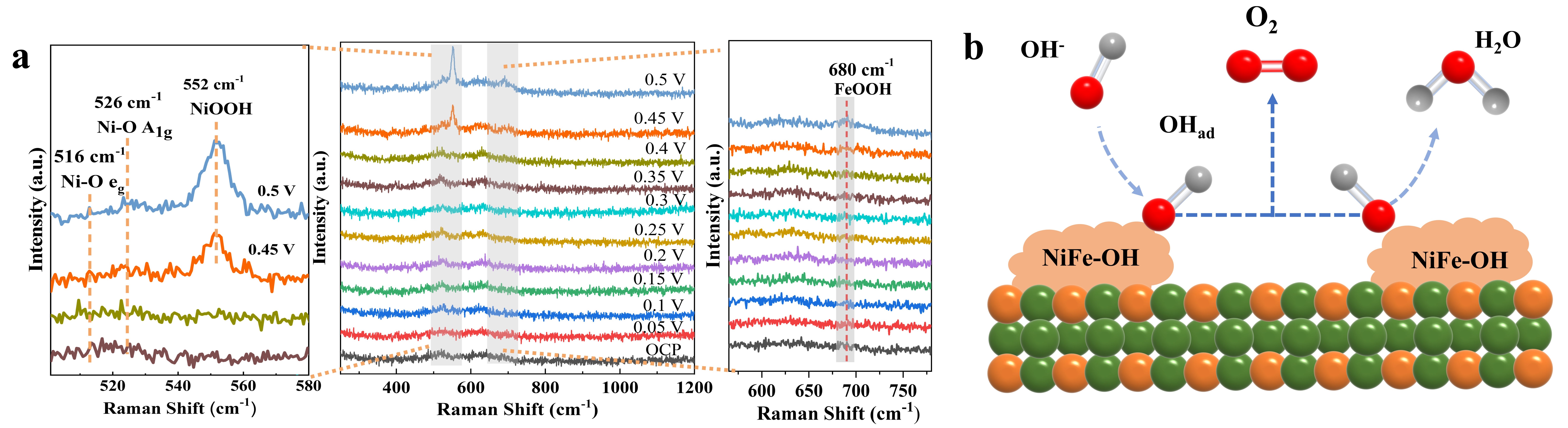


**Fig. S12** (a) In-situ Raman spectra of the Ni-Fe@C_1D&2D_-NO_3_^-^ collected at different potential ranges of OER; (b) Schematic representation of the reactions of OER processes.


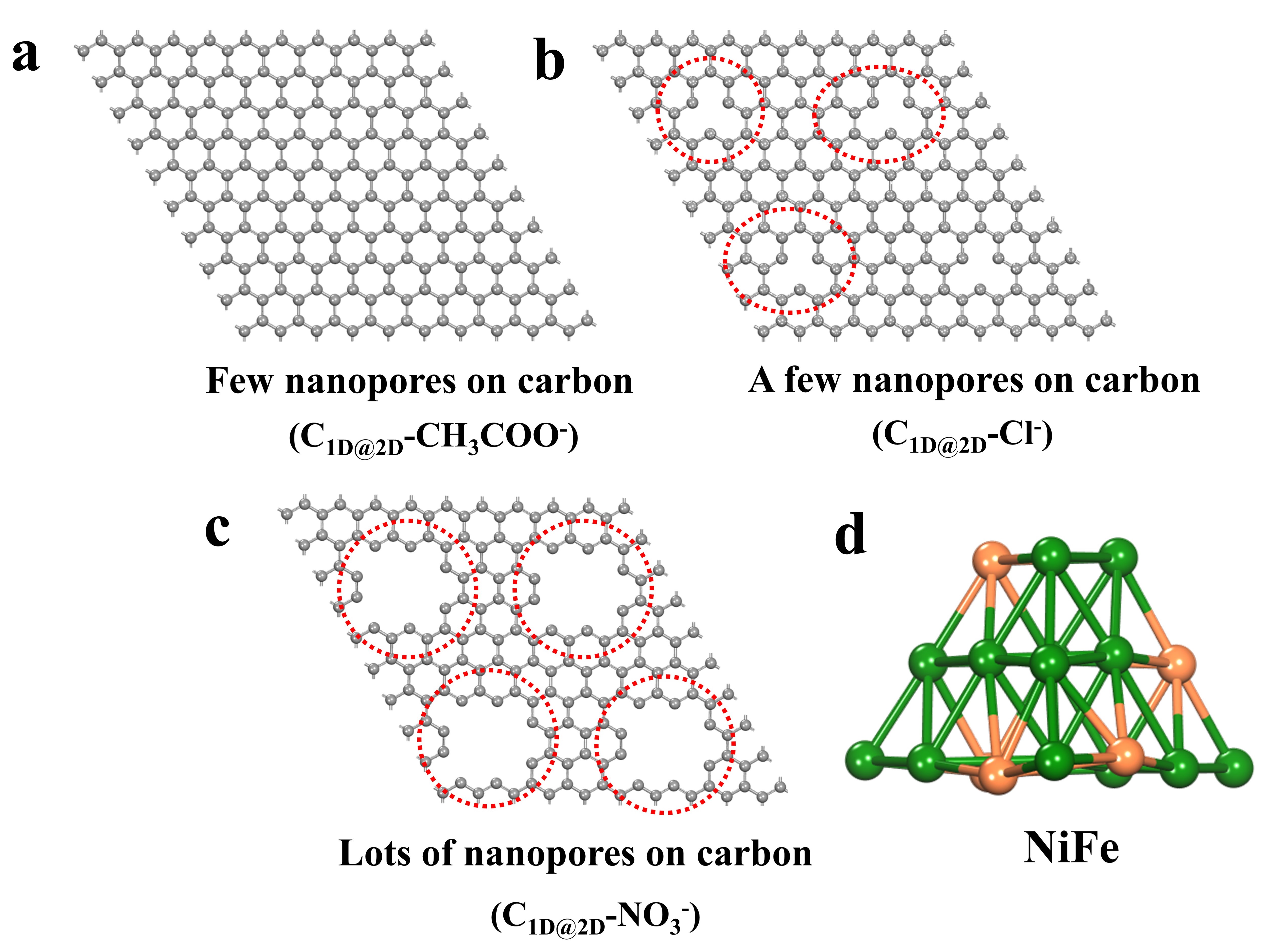


**Fig. S13** Calculation models of (a~c) three kinds of carbon sheets with different defect sizes and (d) Structural configuration of NiFe nanoparticles.


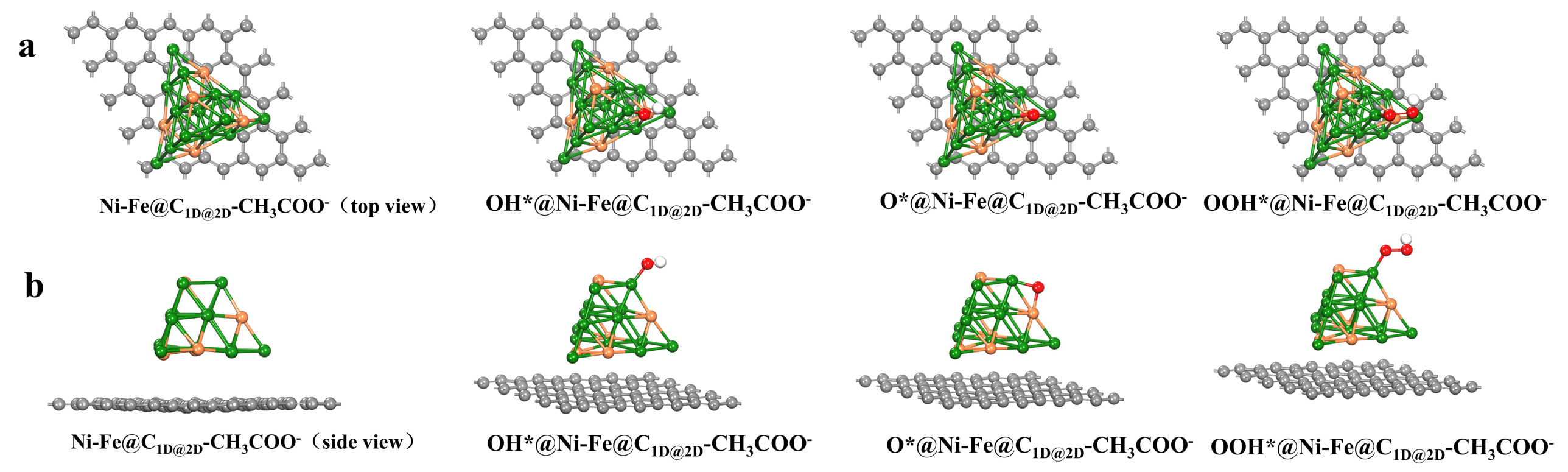


**Fig. S14** Optimized configurations of the intermediates (OOH*, O*, and OH*) adsorbed on Ni-Fe@C_1D@2D_-CH_3_COO^-^: (a) top view and (b) side view.


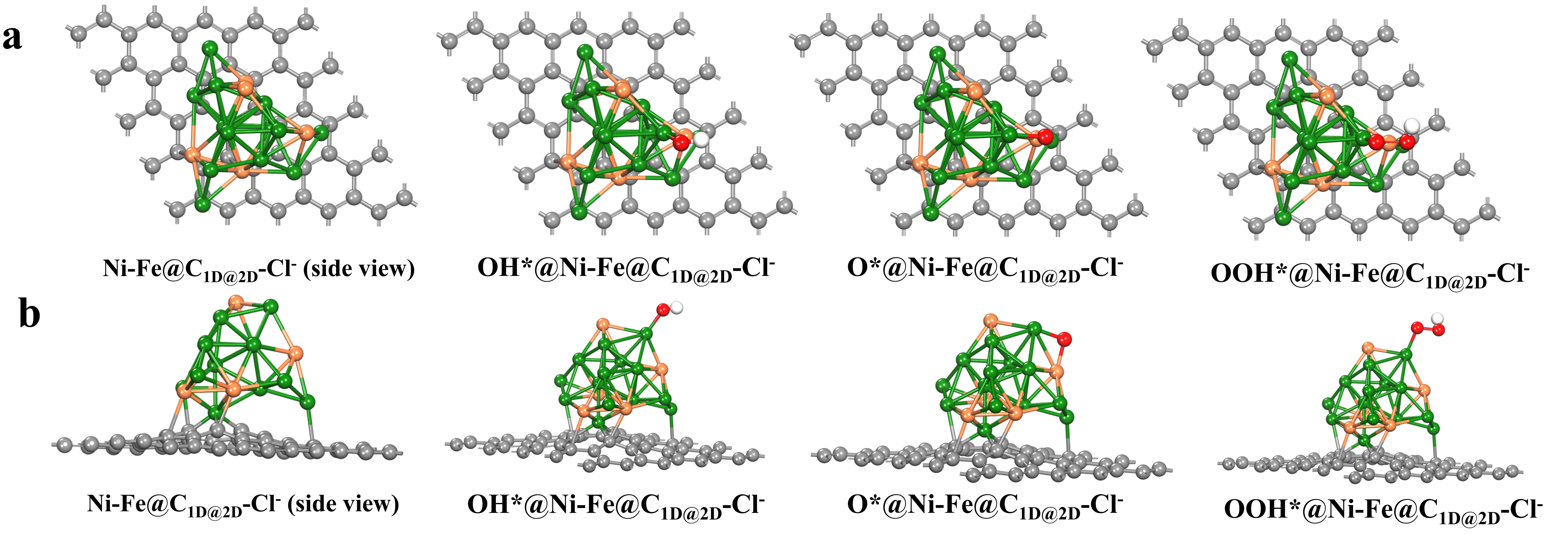


**Fig. S15** Optimized configurations of the intermediates (OOH*, O*, and OH*) adsorbed on Ni-Fe@C_1D@2D_-Cl^-^: (a) top view and (b) side view.

**Tab. S1** BET surface area, pore volume and pore size of the different samples.

| **Sample** | **S_BET_ (m^2^/g)** | **Pore volume (cm^3^/g)** | **Pore size (nm)** |
| --- | --- | --- | --- |
| **Ni-Fe@C_1D&2D_-Cl^-^** | 333.8 | 0.2 | 4.2 |
| **Ni-Fe@C_1D&2D_-CH_3_COO^-^** | 266.6 | 0.5 | 5.5 |
| **Ni-Fe@C_1D&2D_-NO_3_^-^** | 358.7 | 0.5 | 4.6 |

**Tab. S2** Comparison of the OER activity of Ni-Fe@C_1D&2D_-NO_3_^-^ porous networks with recently-reported solid-state catalysts for OER in 1 M KOH electrolyte.

|  | Current density (j mA/cm^2^) | Tafel  (mV/dec) | Overpotential (mV) | Reference |
| --- | --- | --- | --- | --- |
| **Ni-Fe@C_1D&2D_-NO_3_^-^** | **10** | **65.2** | **165** | **This work** |
|  | **40** | **65.2** | **270** | **This work** |

| Fe_2_O_3_@CeO_2_–OV | 10 | 58 | 172 | *Energy Environ. Sci.* **2024**, *17*, 5260 |
| --- | --- | --- | --- | --- |
| W-NiS_0.5_Se_0.5_ | 10 | 41 | 176 | *Adv.Mater.* **2022**, 34, 2107053 |

| NiFe_2_O_4_ | 10 | 62.6 | 180 | *J. Am. Chem. Soc.* **2023**, *145*, 24218 |
| --- | --- | --- | --- | --- |
| Ru-FeNi@NLC | 10 | 72 | 198 | *Angew. Chem. Int. Ed.***2023**, *62*, e202306333 |
| NiFc′Fc/NF | 100 | 45 | 213 | *Angew. Chem. Int. Ed.***2023**, *62*, e202311909 |
| MoZnFeCoNi | 10 | 48.78 | 221 | *Sci. Adv.* **2024**, *10*, eadq6758 |
| (FeCoNiCrCu)Se_x_ | 10 | 55.5 | 222 | *Joule* **2024**, *8*, 2342 |
| Ru-Ni(OH)_2_ | 10 | 55.2 | 228 | *Adv. Mater*. **2024**, *36*, 2403151 |
| 1D NiFeO_x_-P | 10 | 27.07 | 237 | *Angew. Chem. Int. Ed.* **2023**, *62*, 2309732 |
| CoOOH/Co_9_S_8_ | 10 | 86.4 | 240 | *Angew. Chem. Int. Ed.* **2022***,* *61*, e202117178 |
| ZnCo_1.25_Ni_0.73_O_x_-SO_4_ | 10 | 41.6 | 252 | *Appl. Catal. B Environ.* **2023**, *320*, 121988 |
| FeCo_3_(DDA-CH_3_)_2_ | 10 | 46.86 | 260 | *Adv. Mater.* **2024**, *36*, 2402388 |
| Ni35% /MnFe_2_O_4_ | 10 | 38.3 | 261 | *Adv. Mater.* **2024**, *36*, 2400572 |
| P-Ce SAs@CoO | 10 | 75 | 261 | *Adv. Mater.* **2023**, *35*, 2302462 |
| Ni_2_P-CoCH/CFP | 10 | 36 | 270 | *Angew. Chem. Int. Ed.* **2023**, *62*, e202302795 |
| Fe-NiO/NiS_2_ | 10 | 40 | 270 | *Angew. Chem. Int. Ed.* **2022***, 61*, e202207217 |
| Ce-NiCo_2_O_4_ | 10 | 85 | 270 | *Angew. Chem. Int. Ed.* **2024**, e202415306 |
| P-Mo-Co_3_O_4_@CC | 10 | 53.9 | 276 | *Carbon Energy* **2023**, *5*, e279 |
| HO@NiCo-LDH YSMR | 10 | 49.7 | 278 | *Angew. Chem. Int. Ed.* **2022**, *61*, e202213049 |
| CoN | 10 | 89 | 286 | *J. Am. Chem. Soc.* **2024**, *146*, 12556 |
| S/N-CMF@Fe_x_Co_y_Ni_1_-x-y-MOF | 10 | 53.5 | 296 | *Adv. Mater.* **2023**, *35*, 2207888 |
| Mo/C tubes | 10 | 67.3 | 324 | *J. Am. Chem. Soc.* **2022***, 144*, 20571 |
| ZnCo_2_O_4−_*_x_*F*_x_*/CNTs | 10 | 59.2 | 350 | *Angew. Chem. Int. Ed.* **2023**, *62*, e202301408 |
| CoMM | 10 | 84 | 351 | *J. Am. Chem. Soc*. **2023**, *145*, 8052 |
